# Acceleration of Singlet Oxygen Evolution by Sonopiezoelectric Charge Transfer Over SrTiO_3_‐TiO_2_ Heterojunction for Selective Oxidation

**DOI:** 10.1002/EXP.20250012

**Published:** 2026-05-28

**Authors:** Weiwei Wang, Chun Lu, Xiaoxiao Liu, Wenlong Yang, Jie Zhou, Chenyao Hu, Xin Li, Guangze Nie

**Affiliations:** ^1^ School of Environmental Science and Engineering Nanjing Tech University Nanjing People's Republic of China; ^2^ School of Chemistry and Chemical Engineering Nanjing University of Science and Technology Nanjing People's Republic of China; ^3^ Advanced Analysis and Testing Center Nanjing Forestry University Nanjing People's Republic of China; ^4^ Singapore Membrane Technology Centre Nanyang Environment and Water Research Institute Nanyang Technological University Singapore Singapore

**Keywords:** selective oxidation, singlet oxygen, sonopiezo‐catalysis, SrTiO_3_‐TiO_2_, S‐scheme heterojunction

## Abstract

Sonopiezo‐mediated heterogeneous catalysis is a promising technology for efficient removal of organic pollutants from wastewater. Nevertheless, it is challenging in real water matrix with coexisting ions due to the rather‐limited selectivity of reactive oxygen species if any. Herein, a sonopiezo‐triggered singlet oxygen evolution system for selective removal of tetracycline hydrochloride (TCH) was rationally designed by SrTiO_3_‐TiO_2_ heterojunction with a TiO_2_‐terminated SrTiO_3_ S‐scheme interfacial structure. SrTiO_3_‐TiO_2_ heterojunction exhibits a dramatically expanded piezoelectric constant (*d*_33_), which produces a synergistic effect on improving the separation and transfer efficiency of charge carrier for efficient TCH degradation. Moreover, SrTiO_3_‐TiO_2_ heterojunction presents a high sonopiezo‐current, which attractively indicates that SrTiO_3_‐TiO_2_ heterojunction has a potential capacity of charge separation under sonopiezo initiation. Remarkably, the mechanism was identified as the continuous generating of singlet oxygen (^1^O_2_), in which the sonopiezo‐generated electrons trigger the formation of superoxide anion radical (•O_2_^−^) and its subsequent transformation to generate ^1^O_2_ by expending more sonopiezo‐generated holes. These findings provided new insights towards developing nonradical system for effective and selective oxidation of electron‐rich pollutants via sonopiezo‐catalytic process.

## Graphical abstract

A sonopiezo‐triggered singlet oxygen evolution system by S‐scheme SrTiO_3_‐TiO_2_ heterojunction was rationally designed to selectively remove electron‐rich pollutants from water.

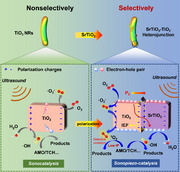

## 1 Introduction

The widespread and inappropriate usage of antibiotics have caused a significant global concern, which have been categorized as emerging contaminants in various environmental matrices even at lower concentrations [1, 2]. Generally, most of antibiotic molecules have more or less electron‐rich structures than other organic pollutants, which potentially elicited a strong toxicity and persistent environmental implications, posing serious challenges for water or wastewater treatment [3]. Although advanced oxidation processes (AOPs) have been developed and demonstrated as powerful methods to effectively remove a variety of pollutants from water by various radical oxidation systems, such as hydroxyl radical (•OH), superoxide anion free radical (•O_2_^−^), and sulfate radical (SO_4_^•−^). However, the radical‐mediated AOPs are suffering from the lower generation, utilization efficiency, and rather‐limited selectivity of above short‐lived radicals, which further hindered their applications in wastewater treatment. Recently, the singlet oxygen (^1^O_2_) dominated AOPs have been widely studied due to its selectivity and anti‐interference ability even in the presence of a complex water matrix [4]. In particular, ^1^O_2_ with electrophilic properties exhibits a good selectivity for electron‐rich organic pollutants, which potentially could be employed to complete the elimination of antibiotics from water. Therefore, it is vital important to facilitate the generation of ^1^O_2_ in AOPs.

Many efforts have been made to regulate the generation of ^1^O_2_ in the AOPs, such as photocatalysis [5], electrocatalysis [6], sonocatalysis [7], and piezocatalysis [8]. Of all, ultrasound‐induced sonocatalysis and piezocatalysis (it is called as sonopiezo‐catalysis) are recently garnered tremendous research exploits on the generation of ROS for environmental remediation [9], antibacterial therapy [10], tumor therapy [11], and hydrogen peroxide (H_2_O_2_) production [12], due to its outstanding effects of microjet [13], shock wave, thermal cavitation [14], sonoluminescence [2], and piezoelectricity [15]. Interestingly, the effect of continuous ultrasonic cavitation in heterogeneous solution not only produces a set of consequent sonochemical effects (sonopiezo‐catalytic process) in semiconductor materials [16], due to the nucleation, growth, and violent collapse of cavitation bubbles, but also leads to the piezoelectric effects (piezocatalytic process) in piezoelectric materials [15]. The main difference between sonopiezo‐catalysis and piezocatalysis is the generation of cavitation, which not only induces piezoelectric polarization but causes transient cavitation [17]. In sonopiezo‐catalytic process, the cavitation effects renders the generation, trapping, and recombination of charge carriers in semiconductor catalysts (e.g. TiO_2_ [18], ZnO [19], ZrO_2_ [20], et al.), subsequently the interfacial charge transfer triggering ROS generation. Piezocatalytic process is another kind of physical phenomenon, in which the semiconductor materials with non‐centrosymmetric structures (e.g. ZnO [21], BaTiO_3_ [22], SrTiO_3_ [23], etc.) can directly convert ultrasonic mechanical energy into chemical energy by the charge carriers induced by external stress. Generally, the generation and transportation of charge carriers (electron–hole pairs, e^−^–h^+^ pairs), play a vital important role in the generation efficiency of ROS [24], which enables the complete degradation of pollutant molecules from wastewater without any secondary pollution [25]. Unfortunately, the bound e^−^–h^+^ pairs both in sonopiezo‐catalytic process and piezocatalytic process tend to be recombined, producing a low catalytic efficiency in environmental remediation, due to strong Coulomb force and dielectric screening of traditional single‐component semiconductors [26].

Generally, the formation of sono‐cavitation can regulate crystallinity and phase of bulk catalysts, altering the deformation and charge distribution of surface crystals with piezoelectric property [27, 28]. Thus, the high‐efficiency generation of ROS is determined by the sono‐cavitation induced the intrinsic charge transfer either in the bulk or at the interfacial or surface of catalysts [29]. Recently, the design and engineering of step‐scheme (S‐scheme) heterojunction cocatalyst is regarded as a promising strategy for boosting the charges transfer and/or excitons e^−^–h^+^ pairs [26]. Significantly, as a kind of ABO_3_‐type perovskite, SrTiO_3_ with a strong piezoelectric strain coefficient (d_33_ ≈ 69.6 pm/V) was always used as co‐catalysts to efficiently accelerate the charge transfer, hinder the charge recombination [30], and further stimulate the high‐efficiency generation of ROS, due to its non‐centrosymmetric polar crystal structure [31]. When it was combined with semiconductor‐based photocatalyst, the photogenerated e^−^ or h^+^ can be captured, and the unfavorable recombination of e^−^–h^+^ pairs can be well prevented via an internal electric field (IEF), due to the formation of S‐scheme n‐n [32] or n‐p [33] junctions. For example, the assistant of SrTiO_3_ is favorable to generate surficial polarizing potential, modulate, and boost the charge transfer trajectory in the type II, Z‐scheme or S‐scheme Schottky/heterojunction, such as SrTiO_3_/TiO_2_ [34] and NH_2_‐MIL‐125(Ti)/SrTiO_3_ heterojunction [35], which exhibit a high efficiency of photocatalytic pollutants degradation or hydrogen production. Meanwhile, the effective utilization of free e^−^ and h^+^ could also be promoted by the considerable polarization electric field (Pz) of SrTiO_3_ nanoparticle [35, 36]. What's more, the coupled piezoelectric SrTiO_3_ with piezoelectric BaTiO_3_ in SrTiO_3_‐BaTiO_3_ heterojunction can significantly promote the generation of piezoelectric charges (q^+^ and q^–^) in the opposite direction of SrTiO_3_ and BaTiO_3_ [37]. These piezoelectric charges not only can promote the rapid separation of free e^−^–h^+^ pairs, but also allows the spontaneous transfer of e^−^ from BaTiO_3_ to SrTiO_3_, which in turn facilitate redox reactions [32]. However, to date, there is few report on facilitating the generation of ^1^O_2_ by modulating the charge transfer trajectory of traditional semiconductor or piezoelectric materials in sono‐cavitation system, especially in material design by S‐scheme heterojunction engineering.

Herein, the unique SrTiO_3_‐TiO_2_ S‐scheme heterojunction was prepared via hydrothermal process and its sonopiezo‐triggered singlet oxygen evolution system for selective removal of tetracycline hydrochloride (TCH) was systematically investigated. The surface atomic structures of TiO_2_‐terminated SrTiO_3_ pave the way of charge transfer from TiO_2_ to SrTiO_3_, further accelerating the rapid separation of free e^−^–h^+^ pairs by the polarization electric field and internal electric fields. More importantly, the intrinsic activity of the optimal SrTiO_3_‐TiO_2_ heterojunction is boosted by the role of S‐scheme heterostructural interface, which artificially tailors an expanded piezoelectric constant (*d*_33_) of 43.5 pm V^−1^ and a high sonopiezo‐current of about 1.5 µA cm^−1^, producing a synergistic effect on improving the separation and transfer efficiency of charge carrier. This attractive change contributes to the continuous generation of ^1^O_2_. These findings provided new insights towards developing nonradical system for effective and selective oxidation of electron‐rich pollutants via sonopiezo‐catalytic process.

## 2 Experimental Section

### 2.1 Materials

All chemical reagents utilized in the experiments were at analytical purity without any purification. Tetracycline hydrochloride (TCH, 96.0%), norfloxacin (NOR, 98%), ciprofloxacin (CIP, 98.0%), azithromycin (AZM, AR), TiO_2_ (anatase, 99.8%), formic acid (HA, HPLC), p‐benzoquinone (p‐BQ), furfuryl alcohol (FFA, AR), 2,2,6,6‐tetramethylpiperidine (TEMP, ≥98.0%), and 5, 5‐dimethyl‐1‐pyrroline oxide (DMPO) were supplied by the Aladdin Reagent Co., Ltd). Amoxicillin (AMO, ≥99.0%), hydrochloric acid (HCl), sodium hydroxide (NaOH, AR), sodium sulfate (Na_2_SO_4_, 99.0%), sodium chloride (NaCl, AR), strontium chloride hexahydrate (SrCl_2_ 6H_2_O, AR), sodium oxalate(Na_2_C_2_O_4,_ AR), methanol (MeOH, AR) and acetonitrile (HPLC) were purchased from Sinopharm Chemical Reagent Co., Ltd. Fluorine‐doped tin oxide (FTO) conductive glasses were purchased from Sigma Aldrich.

### 2.2 Synthesis of SrTiO_3_‐TiO_2_ Heterojunction

**FIGURE 1:**
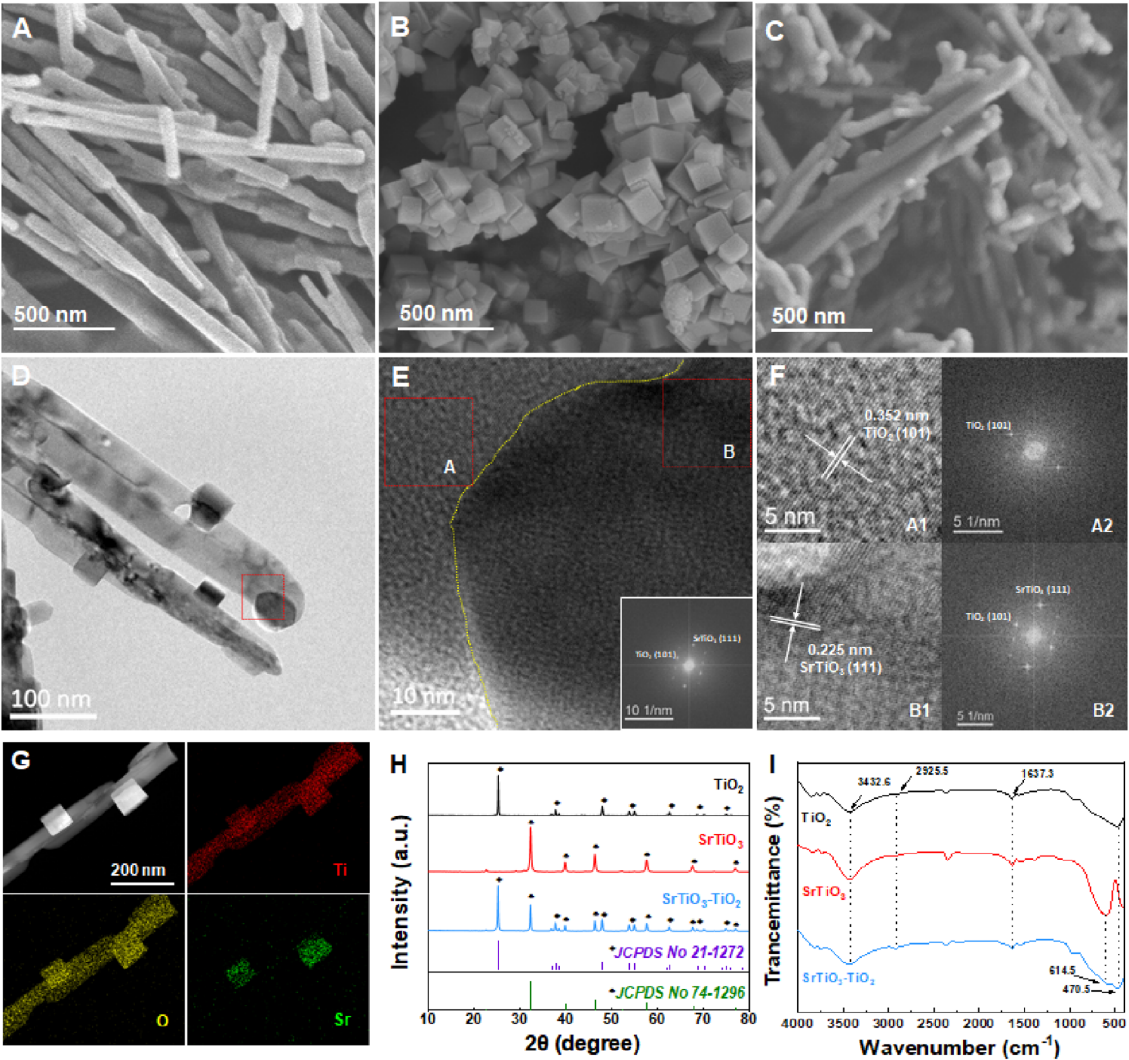
SEM images of (A) TiO_2_ NRs, (B) SrTiO_3_ NPs, and (C) SrTiO_3_‐TiO_2_ heterojunction. (D) TEM and (E) HRTEM images of SrTiO_3_‐TiO_2_ heterojunction and the corresponding FFT patterns (insert image). (F) Lattice fringe images and corresponding FFT patterns of marked A and B insert in (E), respectively. (G) EDX elemental mapping of SrTiO_3_‐TiO_2_ heterojunction. XRD patterns (H) and FT‐IR spectrum (I) of TiO_2_ NRs, SrTiO_3_ NPs, and SrTiO_3_‐TiO_2_ heterojunction.

SrTiO_3_‐TiO_2_ heterojunction was prepared by a two‐step hydrothermal method. The proof‐of‐concept design of SrTiO_3_‐TiO_2_ heterojunction is proposed, in which TiO_2_ nanorods (TiO_2_ NRs) are used as a typical semiconductor sonocatalyst to support the growth of TiO_2_‐terminated SrTiO_3_ nanoparticles, as illustrated in Figure 1. Initially, the TiO_2_ NRs precursor was synthesized by hydrothermal pretreating TiO_2_ nanoparticles (TiO_2_ NPs, 3.0 g) with NaOH solution (50 mL, 10 m) for 24 h at 180°C in Teflon‐lined autoclave using as raw materials. The final TiO_2_ NRs were obtained after HCl acidizing and subsequently calcining at 700°C, according the reported method [38]. Then, the SrTiO_3_‐TiO_2_ heterojunction was synthesized by integrating SrTiO_3_ nanoparticles on the surface of TiO_2_ NRs via a hydrothermal method.

A series of SrTiO_3_‐TiO_2_ heterojunction composites with different molar ratio of SrTiO_3_ and TiO_2_ were prepared. Typically, the amount of TiO_2_ NRs was added to NaOH solution containing SrCl_2_ (molar ratio of Sr and Na = 0.5) under continues agitation until producing a white and uniform colloidal mixture system, and the molar ratio of Sr and Ti was tuned as 0.05, 0.1, 0.15, 0.2, 0.3, and 0.4, respectively. Then, the mixture was transferred in a 200 mL Teflon‐lined autoclave and heated at 180°C for 12 h. The resultant samples were obtained after washing and drying, and they were named as SrTiO_3_‐TiO_2_‐X (X = 1/2/3/4/5/6, corresponding with the molar ratio of Sr and Ti with 0.05/0.1/0.15/0.2/0.3/0.4). For the sake of simplicity, SrTiO_3_‐TiO_2_‐4 was determined as a representative of SrTiO_3_‐TiO_2_ heterojunction unless otherwise stated. Meanwhile, for comparison, SrTiO_3_ NPs were prepared by mixing TiO_2_ NPs (0.25 g) and SrCl_2_ in 150 mL NaOH solution (molar ratio of Sr and Na = 0.5) in a 200 mL hydrothermal Teflon‐lined autoclave for 24 h at 180°C to obtain SrTiO_3_ NPs.

### 2.3 Characterization Methods

Scanning electron microscopy (SEM, Hitachi Regulus 8100) and Transmission electron microscopy (TEM, JEM2100) were used to determine the surface morphology and structure of SrTiO_3_‐TiO_2_ heterojunction. The crystal structure and composition of SrTiO_3_‐TiO_2_ heterojunction were characterized by using X‐ray diffractometer (XRD, MiniFiex600) with Cu Kα radiation at 40 kV and 40 mA. X‐ray photoelectron spectroscopy (XPS, Thermo Fisher Scientific K‐Alp) was conducted to analyze the electronic states and chemical composition of SrTiO_3_‐TiO_2_ heterojunction. The valence band spectra (VB‐XPS) of the samples was determined by X‐ray photoelectron spectroscopy (XPS, PHI5000 VersaProbe). The total content of SrTiO_3_ in heterojunction was detected by Inductive coupled plasma (ICP, Focused Photonics Inc. ICP‐5000). The surface groups of samples were analyzed by the Fourier transform infrared spectroscopy (FT‐IR, Gangdong‐650S).

The piezoelectric properties of SrTiO_3_‐TiO_2_ heterojunction were investigated by using a piezo‐response force microscope (PFM, Bruker Dimension Icon) with an alternating voltage ranging from −10∼10 V, at a predetermined scanning path. To obtain the piezoelectric strain coefficients (*d*_33_) of SrTiO_3_‐TiO_2_ heterojunction, the signals of morphological, phase, and amplitude corresponding with the local deformation and polarization state of the samples were collectively recorded under the electric field. The specific energy level including band gaps and flat‐band potentials of samples were determined by the ultraviolet–visible diffuse reflectance spectra (UV‐DRS) and Mott–Schottky (MS) curves, respectively. UV‐DRS of samples were recorded by using UV–visible spectrophotometer (UV, PerkinElmer Lambda 950) with an absorption wavelength ranging from 200∼600 nm. Ultraviolet photoelectron spectroscopy (UPS, Thermo Fisher Scientific ESCALAB XI+) was used to determine the work function (Φ) of the sonopiezo‐catalysts. MS curves, transient piezoelectric current and the electrochemical impedance spectroscopy (EIS) were measured using CS350 Electrochemical Workstation (Wuhan Corrtest Instrument Corp., Ltd). Three electrode system was employed, in which the silver/silver chloride (Ag/AgCl) and platinum foil (Pt) electrodes were served as the reference electrode and the counter electrode, respectively. A 0.25 M Na_2_SO_4_ solution was used as the electrolyte. As‐prepared samples were employed as the working electrode, which was prepared by uniformly coating 4 mg of catalyst and 10 µL of Nafion solution on a 1×1 cm FTO glass.

The generated active species (•OH, h^+^, •O_2_^−^, and ^1^O_2_) were respectively determined by a series of quenching experiments and using spin trapping electron paramagnetic resonance (EPR, Bruker 5000). In the quenching experiments, MeOH [39], Na_2_C_2_O_4_ [40], p‐BQ [41], and FFA [42] were respectively used as scavengers agent to quench the generated •OH, h^+^, •O_2_^−^, and ^1^O_2_ from synergic sonocatalytic and piezocatalytic degradation system of TCH. Typically, 1∼5 mM^−1^ of MeOH, Na_2_C_2_O_4_, p‐BQ, and FFA were respectively added in 200 mL of TCH solution. For radical trapping experiments, TEMP and DMPO were used to identify ^1^O_2_ and •OH, respectively. Typically, 100 µL of the sample (1 mg mL^−1^) was mixed with 10 µL of TEMP (1 mg mL^−1^) or DMPO solution (100 mM) in 1.5 mL sample tube with and without ultrasound irradiation.

### 2.4 Sonopiezo‐Catalytic TCH Degradation Performance

**SCHEME 1:**
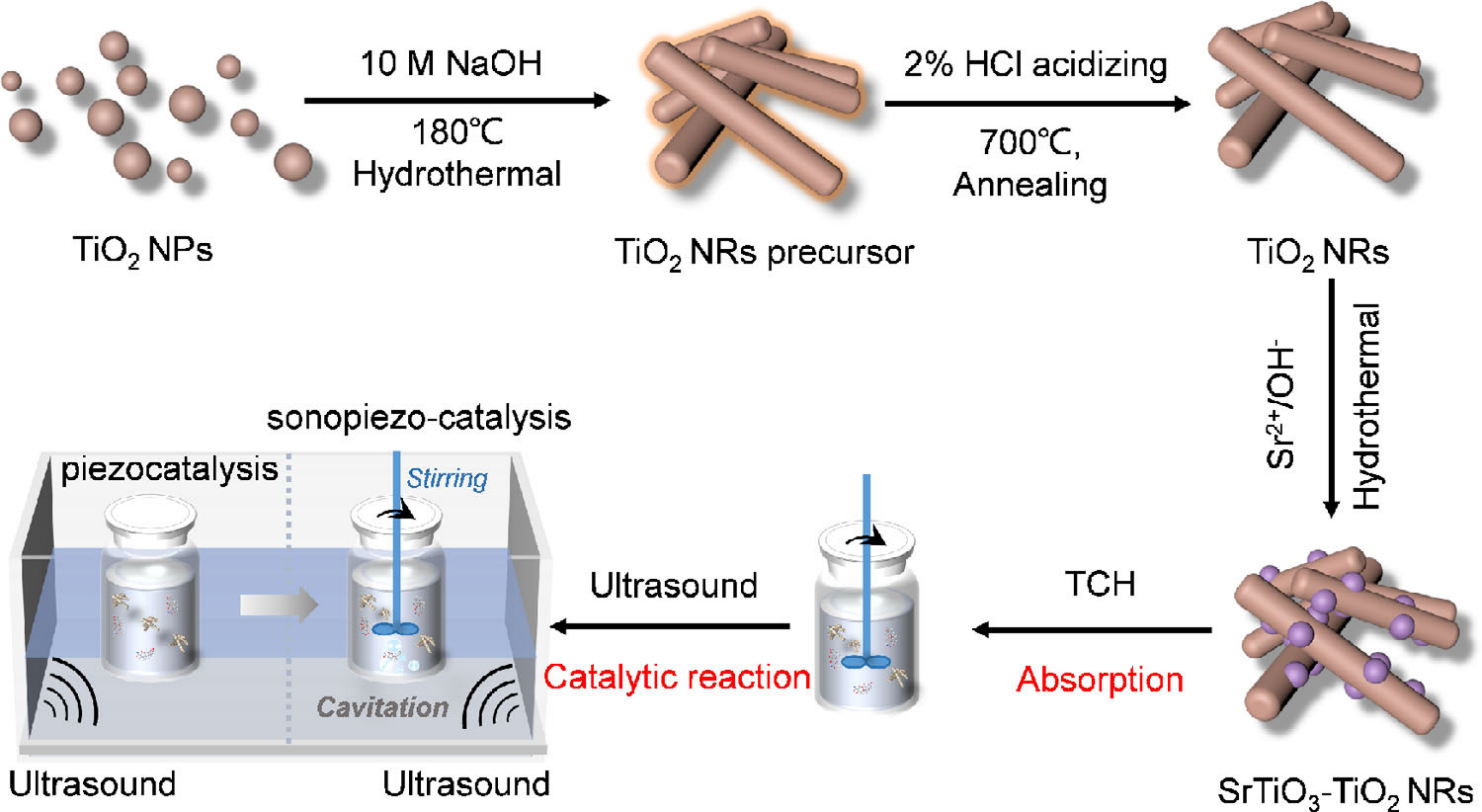
Schematic illustration of the synthesis route and the sonopiezo‐catalytic reactor diagram of SrTiO_3_‐TiO_2_ heterojunction.

The adsorption and catalytic reaction experiments were conducted in the sonopiezo‐catalytic reactor as illustrated in Scheme 1 and Figure S1. After adsorption equilibrium, the pizeocatalytic and sonopiezo‐catalytic performances of as‐prepared TiO_2_ NRs, SrTiO_3_ NPs, and SrTiO_3_‐TiO_2_ heterojunction were determined in simulated wastewater containing trace amounts of TCH, under ultrasound irradiation. To explore the sonopiezo‐catalytic and piezocatalytic performance of SrTiO_3_‐TiO_2_ heterojunction, the degradation efficiency of several organic pollutants was measured under ultrasonic vibration at 300 W and 45 kHz, and the ultrasonic power density calculations is 0.26 W/cm^2^ (KQ‐500VDV). The degradation of TCH was investigated under sonopiezo‐catalytic process. In the experiments, 0.5 g L^−1^ of powder catalysts were initially dispersed in TCH solution (100 mL, 10 mg L^−1^). Then, the adsorption‐desorption equilibrium between the catalyst and organic molecules was ensured at room temperature under magnetic stirring for above 60 min. After that, the above suspension was transferred to the sonopiezo‐catalytic reactor. And the continuous circulated cooling water was employed to keep the temperature of the ultrasonic bath constant at below 25°C. During the sampling process, 2.0 mL or 1.0 mL of suspension were periodically collected from the reactor and filtered with a 0.22 µm water‐based filter to separate the catalyst powder. Finally, the concentration of model pollutants (TCH, CIP, and OFX) was determined by UV–vis spectrophotometer (N4s, Shanghai Yidian). And the concentration of AMO and AZM was analyzed by high performance liquid chromatography (HPLC, Agilent 1200) equipped with Kinetex C18 (5.0 µm, 150.0 × 4.6 mm column). For AMO detection, methanol: 1% formic acid in water was used as HPLC mobile phases (20:80, v/v) and the quantification of AMO was achieved with UV detection at 230 nm. For quantification of AZM, acetonitrile: KH_2_PO_4_ solution (25 mM) was performed with mobile phase (30:70, v/v) and the UV detection was set at 200 nm.

## 3 Results and Discussion

### 3.1 Characterization of SrTiO_3_‐TiO_2_ Heterojunction

The surface morphology of TiO_2_ NRs, SrTiO_3_ NPs, and SrTiO_3_‐TiO_2_ heterojunction was observed using scanning electron microscopy (SEM), as shown in Figure 1A–C. We performed the further analysis on the average length and diameter of TiO_2_ nanorods as well as SrTiO_3_ nanoparticles employed by Nano measurer software (Figure S2). Results show that pure TiO_2_ sample consists of uniform nanorods with an average diameter of approximately 62 nm and an average length of 842 nm, while pure SrTiO_3_ is composed of irregular nanoparticles with an average diameter of 103.7 nm, it is close to a tetrahedral morphology. As shown in Figure 1C, SrTiO_3_‐TiO_2_ heterojunction remained the rod‐like morphology of TiO_2_ NRs coated with SrTiO_3_ NPs. Furthermore, the high‐resolution TEM (HRTEM) and EDX elemental mapping were employed to reveal the detailed surface atomic structures (Figures 1D–G and Figure S3). It presents the TEM and HRTEM images of pure SrTiO_3_ NPs and pure TiO_2_ NRs. It was observed that pure TiO_2_ NRs exhibits a unique lattice fringe of 0.352 nm, which is in good agreement with the (101) planes of the anatase TiO_2_ phase (Figure S3A–C). At the same time, HRTEM image in Figure S3D–F confirmed that the pure SrTiO_3_ NPs shows well resolved lattice fringe of 0.391 and 0.225 nm, which are corresponding to the (111) and (100) crystal planes of cubic phase of SrTiO_3_ perovskite, respectively. By comparison, SrTiO_3_‐TiO_2_ clearly displays that the roughly SrTiO_3_ NPs were homogeneously deposited on the surface of TiO_2_ NRs. Notably, it was observed that the interface of a phase junction between SrTiO_3_ NPs and TiO_2_ NRs can be identified (inset of Figure 1E). The detailed lattice fringes and interplanar spacings corresponding to the dotted square area marked A and B were further analyzed, as shown in Figure 1F. Before the formation of SrTiO_3_‐TiO_2_ heterojunctions, only (001) crystal faces were observed on the FFT images of TiO_2_ NRs (Figure S3C). While after the formation of SrTiO_3_‐TiO_2_ heterojunctions, both (101) crystal faces attributing to TiO_2_ NRs and (111) crystal faces representing SrTiO_3_ could be observed in Figure 1E, revealing that the SrTiO_3_ NPs were well patched on the surface of TiO_2_ NRs. Furthermore, the chemical composition of SrTiO_3_‐TiO_2_ heterojunction was confirmed by the energy‐dispersive X‐ray (EDX) spectroscopy elemental mapping [43, 44]. Figure 1G indicates that the elements of Ti and O are homogeneously distributed on the SrTiO_3_ and TiO_2_, while Sr signal is uniformly dispersed throughout the SrTiO_3_ NPs.

To further analyze the crystal structures of TiO_2_ NRs, SrTiO_3_ NPs, and SrTiO_3_‐TiO_2_ heterojunction, XRD analysis was performed. Within the 2*θ* range of 10° to 80°, the TiO_2_ NRs, SrTiO_3_ NPs, and SrTiO_3_‐TiO_2_ heterojunction displayed high crystallization (Figure 1H). The diffraction peaks of TiO_2_ at 25.2°, 37.9°, 48.0°, 53.9°, 62.7°, 70.3°, 75.2° are perfectly attributed to the (101), (001), (200), (105), (204), (116), and (215) crystal planes of anatase TiO_2_ (JCPDS No. 21–1272) [38], indicating the high purity of the TiO_2_ NRs. As for SrTiO_3_ NPs, the diffraction peaks at 22.8°, 32.4°, 40.0°, 46.5°, 57.8°, 67.8°, and 77.2° could be assigned to the (100), (110), (111), (200), (211), (220), and (310) crystal planes of cubic‐phase for SrTiO_3_ (JCPDS No. 74–1296) [45]. Notably, the intense peaks of anatase TiO_2_ and cubic‐phase SrTiO_3_ were observed in SrTiO_3_‐TiO_2_ heterojunction with no signs of any impurity phases and the ample exposure (101) plane of TiO_2_ and (111) plane of SrTiO_3_ were also concurred with previous TEM observation. By comparison, only (100) crystal planes of SrTiO_3_ are detected in SrTiO_3_‐TiO_2_‐1, due to the low SrTiO_3_ content in the composites, while the intensity of the diffraction peaks of SrTiO_3_ increases with the loading of SrTiO_3_ in SrTiO_3_‐TiO_2_ heterojunction, determined by XRD (Figure S4). It indicates that the proportion of SrTiO_3_ in SrTiO_3_‐TiO_2_ heterojunction can be regulated by the molar ratio of Sr and Ti. Additionally, FT‐IR spectra were used to investigate the functional groups on the surface of TiO_2_ NRs, SrTiO_3_ NPs and SrTiO_3_‐TiO_2_ heterojunction, as shown in Figure 1I. The vibrations at 3432.6 and 1637.3 cm^−1^ in TiO_2_ NRs, SrTiO_3_ NPs, and SrTiO_3_‐TiO_2_ heterojunction are attributed to the stretching of O─H bending [46]. Meanwhile, the peaks at around 2925.5 cm^−1^ was attributed to the stretching vibration of C‐H symmetric [47]. While, the peak at 614.5 cm^−1^ is attributed to the TiO_6_ stretching vibration of SrTiO_3_ [48]. The stretching vibrations of Ti─O─Ti bond could be found by the peak at 470.5 cm^−1^ in the spectrum of TiO_2_ [46, 49]. By comparison, the peaks of TiO_2_ and SrTiO_3_ within the wavenumber range of 400 to 750 cm^−1^ all appeared in the SrTiO_3_‐TiO_2_ heterojunction and the peaks in SrTiO_3_‐TiO_2_ heterojunction showed a little shift, confirming successful synthesis of SrTiO_3_‐TiO_2_ heterojunction. Although the epitaxial growth of TiO_2_ can occur on the SrTiO_3_ [50], few research reports the epitaxial growth of SrTiO_3_ can happen to TiO_2_. Based on above results, we found that SrTiO_3_ NPs with a close tetrahedral morphology was well grown on the surface of TiO_2_ NRs, which indicates that the epitaxial growth of SrTiO_3_ on TiO_2_ may be achieved. It could be attributed to the high surface energy of TiO_2_‐terminated (100) facets and SrO_3_^4−^‐terminated (111) facets, leading to the formation of Ti‐O‐Ti between SrTiO_3_ on TiO_2_ in SrTiO_3_‐TiO_2_ heterojunction [51]. Thereby, by a simple two‐step hydrothermal method, it is easily achieved for preparing SrTiO_3_‐TiO_2_ heterojunction.

**FIGURE 2:**
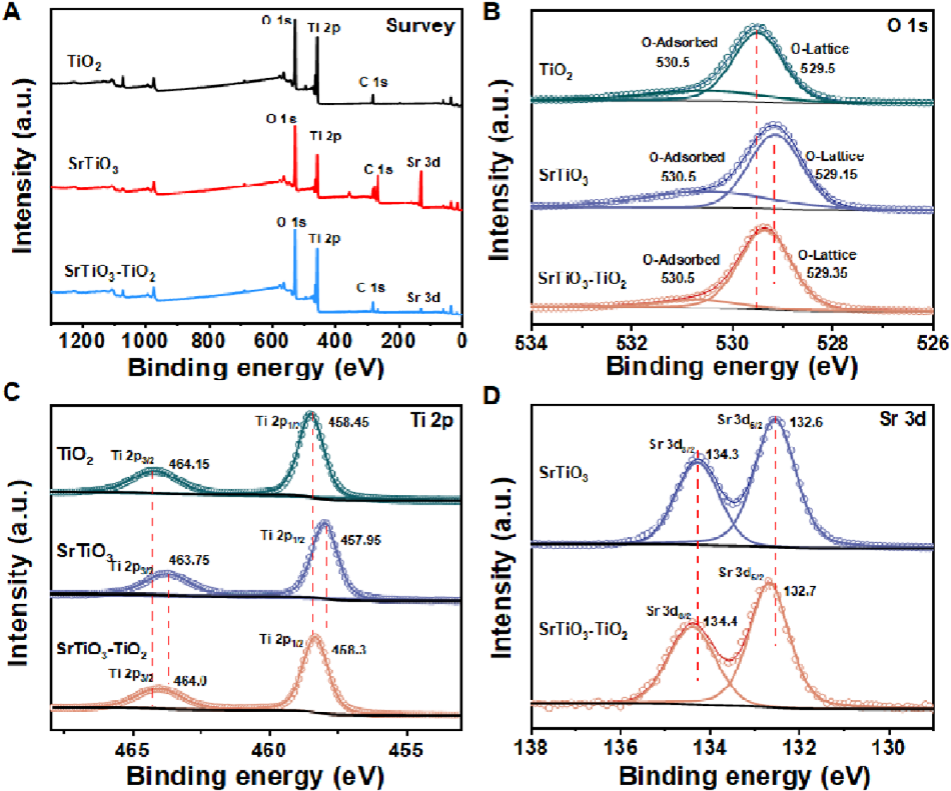
XPS spectra of TiO_2_ NRs, SrTiO_3_ NPs, and SrTiO_3_‐TiO_2_ heterojunction (A) survey, (B) O 1s, (C) Ti 2p, (D) Sr 3d.

To investigate the chemical states and elemental composition of various sonocatalysts, TiO_2_ NRs, SrTiO_3_ NPs, and SrTiO_3_‐TiO_2_ heterojunction were analyzed by XPS. The survey spectra of SrTiO_3_‐TiO_2_ heterojunction confirms the presence of Ti, O, and Sr elements (Figure 2A). For the high‐resolution spectrum of O1s (Figure 2B), the characteristic peak of O 1s could be decomposed into two peaks at 530.5 and 529.5 eV, which are assigned to the surfaced‐adsorbed oxygen and lattice oxygen, respectively [52, 53, 54]. The characteristic peaks of TiO_2_ NRs and SrTiO_3_ NPs at 529.5 and 529.15 eV are attributed to Ti─O─bond and Sr─O─Ti, respectively. In contrast, the introduction of SrTiO_3_ into TiO_2_ NRs results an increased binding energy of lattice oxygen with about 0.15 eV, but it is still lower than that of SrTiO_3_ NPs, indicating that the abundant e^−^ of Sr─O─Ti bond in SrTiO_3_ NPs may flow to TiO_2_ NRs due to the higher electronegativity of Ti atoms (1.54) of TiO_2_ NRs than that of Sr atoms (0.95) after the synthesis of SrTiO_3_‐TiO_2_ heterojunction [55], confirming the important role of heterojunction in the regulation of lattice oxygen. Meanwhile, two sub‐peaks at 464.0 and 458.3 eV were obtained in SrTiO_3_‐TiO_2_ heterojunction, which are respectively attributed to Ti 2p_1/2_ and Ti 2p_3/2_, according to the literature (Figure 2C) [56]. Notably, it is worth pointing out that the different binding energy positions of Ti 2p between TiO_2_ NRs and SrTiO_3_ NPs are observed, which can be attributed to the Ti^4+^ state of TiO_2_ (458.45 eV and 464.15 eV, respectively) and Ti^4+^ state of TiO_3_2^−^ (457.95 eV and 463.75 eV, respectively) [57, 58]. Meanwhile, the binding energies of Ti 2p of SrTiO_3_‐TiO_2_ heterojunction exhibit a slight decreased shift with about 0.15 eV compared with that of TiO_2_ NRs, revealing the effect of heterojunction on the Ti─O chemical bond. Thus, the in situ growth of SrTiO_3_ NPs on TiO_2_ NR favors the formation of heterojunction interface. Figure 2D illustrates the element binding energy of Sr 3d at the peak positions of 134.3 and 132.6 eV, which are ascribed to Sr 3d_3/2_ and Sr 3d_5/2_ of SrTiO_3_ [57]_._ Furthermore, a slight shift in the in SrTiO_3_‐TiO_2_ heterojunction is observed, which demonstrates the intimate interfacial contacts between TiO_2_ NRs and SrTiO_3_ NPs. These results proved that the S‐scheme heterojunction of SrTiO_3_‐TiO_2_ provide a potential platform for electron transferring [59].

**FIGURE 3:**
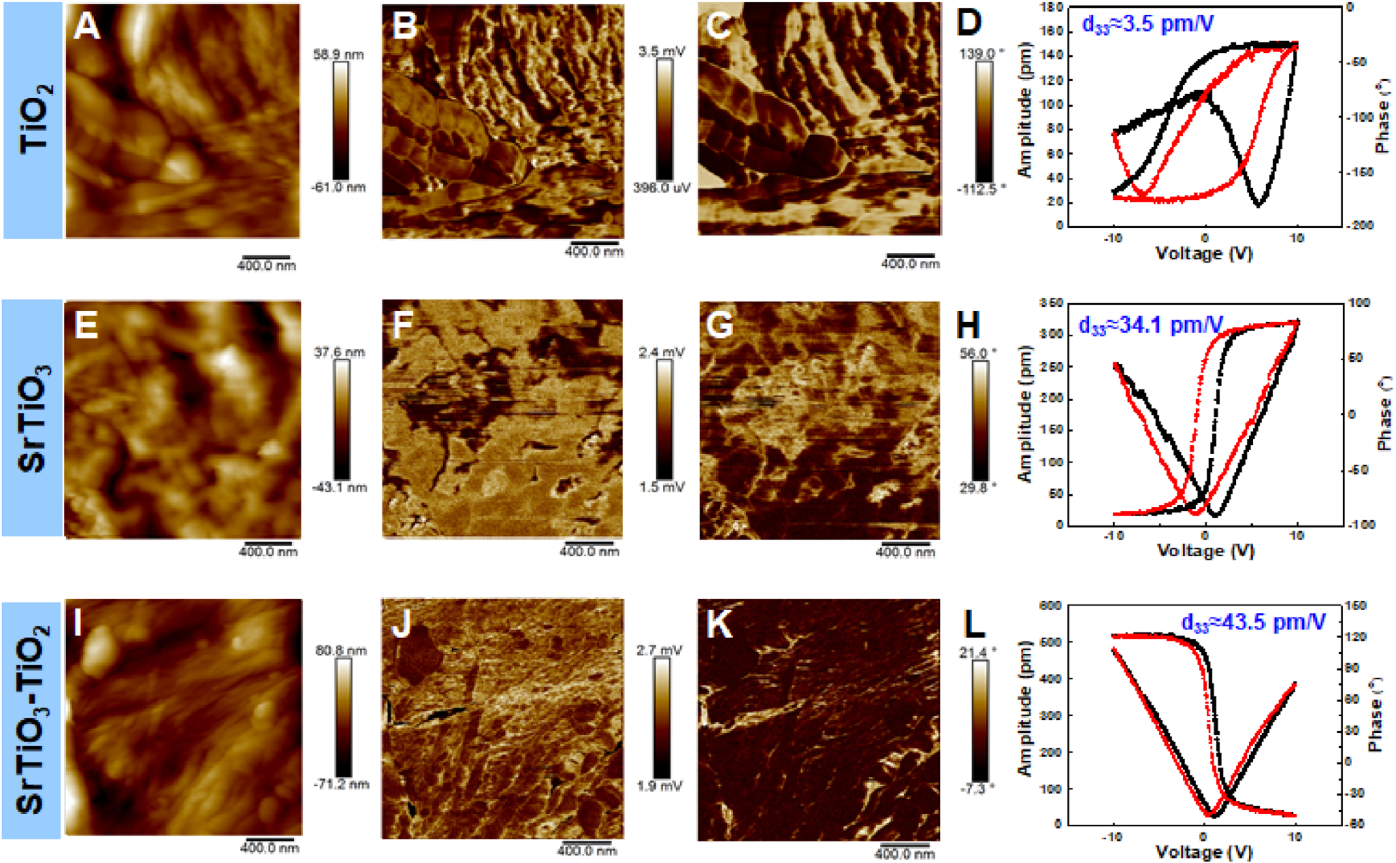
The morphology, amplitude, phase, and amplitude/phase‐voltage curves of (A–D) TiO_2_ NRs, (E–H) SrTiO_3_ NPs, and (I–L) SrTiO_3_‐TiO_2_ heterojunction.

To better determine the piezoelectric properties of samples, the PFM measurement was employed. As demonstrated in Figure 3A,E, when the applied tip voltage was 0 V, the topographies mimic the typical nanorod morphology of TiO_2_ NRs and the typical nanoparticle morphology SrTiO_3_ NPs, respectively, aligning with SEM and TEM observations. By comparison, the topography of SrTiO_3_‐TiO_2_ heterojunction mimics the typical morphology of SrTiO_3_ NPs loaded on the surface of TiO_2_ NRs, in agreement with TEM and EDS mapping observations, as shown in Figure 3I. After applying a deflection voltage of 10 V, the random and clearly visible piezoelectric domains was observed in the bright amplitude diagram of the corresponding materials (Figure 3B,F,J). Furthermore, the phase contrast images clearly exhibited distinct light and dark regions of the TiO_2_ NRs, SrTiO_3_ NPs, and SrTiO_3_‐TiO_2_ heterojunction (Figure 3C,G,K), signifying varying polarization directions within the three catalysts [60]. Furthermore, a typical butterfly amplitude hysteresis loop and well‐defined 180° phase hysteresis loop evidence a piezoelectric response in TiO_2_ NRs, SrTiO_3_ NPs, and SrTiO_3_‐TiO_2_ heterojunction, under a bias field of ± 10 V direct current (Figure 3D,H,L) [47, 61]. Moreover, the butterfly loops of SrTiO_3_‐TiO_2_ heterojunction with a maximum amplitudes of 453.5 pm is higher than that of TiO_2_ NRs (142.3 pm) and SrTiO_3_ NPs (306.2 pm). Based on above results, the effective piezoelectric coefficients (*d*_33_) of samples were calculated by the empirical formula Equation (1): [61]

(1) $$d_{33}=\frac{A-A_{1}}{V-V_{1}}$$

where *V*_1_ and *A*_1_ stand for the voltage and amplitude, respectively, at the intersection of the loop. *V* and *A* stand for their respective values at different points of the loop.

Results showed that the d_33_ values of TiO_2_ NRs and SrTiO_3_ NPs are 3.5 pm V^−1^ and 34.1 pm V^−1^, respectively, which indicates that the TiO_2_ sonocatalyst have low piezoelectric response and the SrTiO_3_ NPs have high piezoelectric response. While the d_33_ value of SrTiO_3_‐TiO_2_ heterojunction is 43.5 pm V^−1^, suggesting that the piezoelectric response is mainly enhanced by the load of SrTiO_3_ NPs and piezoelectric effects should occur at the heterojunction or near the SrTiO_3_ NPs rather than over a large area.

### 3.2 Performance of Sonopiezo‐Catalysis

**FIGURE 4:**
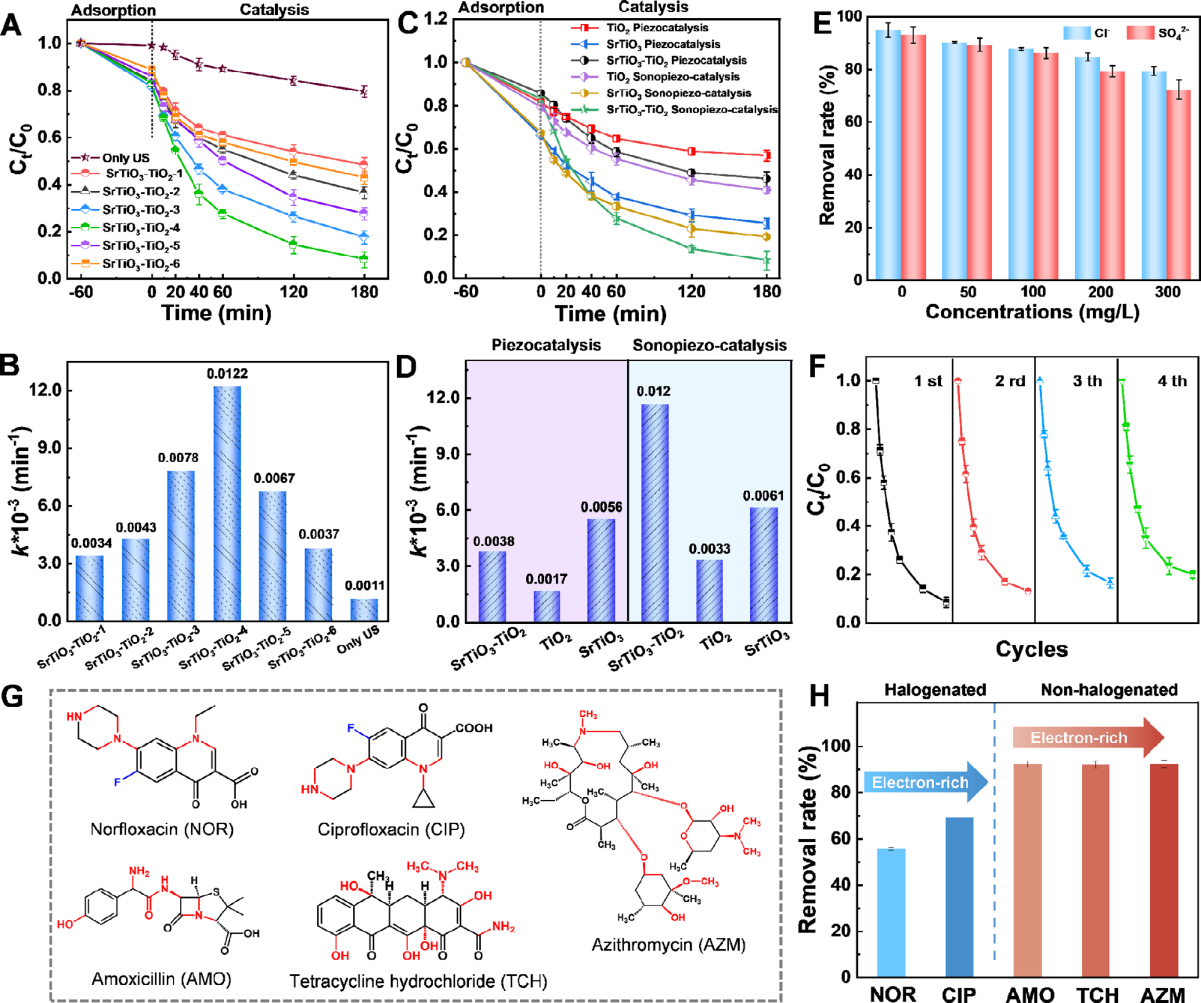
(A) The role of SrTiO_3_ in SrTiO_3_‐TiO_2_ heterojunction for sonopiezo‐catalytic TCH degradation, and (B) the corresponding k constant. (C) Comparison of piezocatalysis and sonopiezo‐catalysis of TiO_2_ NRs, SrTiO_3_ NPs, and SrTiO_3_‐TiO_2_ heterojunction for sonopiezo‐catalytic TCH degradation, and (D) the corresponding k constant. (E) Effects of Cl^−^/SO_4_2^−^ concentrations on TCH degradation by SrTiO_3_‐TiO_2_ heterojunction. (F) Cycling experiment of SrTiO_3_‐TiO_2_ heterojunction for removal of TCH. (G) Structural formulas of typical antibiotic contaminants. (H) Removal rate of various antibiotic contaminants by SrTiO_3_‐TiO_2_ heterojunction.

The sonopiezo‐catalytic and piezocatalytic properties of samples were first investigated by the degradation of TCH under ultrasound condition. Compared with US alone, the TCH degradation efficiency was significantly improved after the addition of sonocatalysts (Figure 4A), indicating that the US‐based heterogeneous AOPs play an important role in the TCH degradation in the present of SrTiO_3_‐TiO_2_ heterojunction. Moreover, with the proportion of SrTiO_3_ in SrTiO_3_‐TiO_2_ heterojunction increased, the degradation efficiency of TCH increased from 51.7% (SrTiO_3_‐TiO_2_‐1) to 92.7% (SrTiO_3_‐TiO_2_‐4) and then decreased to 57.1% (SrTiO_3_‐TiO_2_‐6); the reaction rate constant (*k*) displayed a similar trend increasing from 0.0034 min^−1^ to 0.012 min^−1^ before falling to 0.0037 min^−1^ (Figure 4B). It's probably because the overload of SrTiO_3_ NPs in SrTiO_3_‐TiO_2_ heterojunction, leading to the increase of the grain size of SrTiO_3_ NPs. And it further reduces the distribution of TCH on the surface active sites of SrTiO_3_‐TiO_2_ heterojunction [52, 62]. These results reveal the optimal ratio of hetero‐combination and illustrate that the amalgamation of TiO_2_ NRs and SrTiO_3_ NPs enhances the degradation activity by. Subsequently, ICP was used to determine that the mass percentage of SrTiO_3_ in the SrTiO_3_‐TiO_2_‐4 heterojunction was approximately 5 wt%.

In previous studies, Lu et al. [17]. have proposed how to distinguish the piezocatalysis and sonopiezo‐catalysis. Figure 4C illustrates the TCH degradation efficiency of various catalysts when subjected to the piezocatalytic and the sonopiezo‐catalysis. Thus, the comparative experiments were conducted among the TiO_2_ NRs, SrTiO_3_ NPs, and SrTiO_3_‐TiO_2_ heterojunction to verify the synergistic effect between piezocatalysis and sonocatalysis on the removal of TCH. Compared with TCH degradation efficiency of TiO_2_ NRs, SrTiO_3_ NPs, and SrTiO_3_‐TiO_2_ heterojunction in piezocatalysis system, which have improved significantly in sonopiezo‐catalysis system. Additionally, the SrTiO_3_‐TiO_2_ heterojunction exhibited a more superior degradation efficiency of TCH than TiO_2_ NRs and SrTiO_3_ NPs in the sonopiezo‐catalysis system. As shown in Figure 4D, the degradation rate constants of TiO_2_ NRs, SrTiO_3_ NPs, and SrTiO_3_‐TiO_2_ heterojunction under the sonopiezo‐catalysis conditions were higher than that of under the piezocatalysis conditions. The results also showed that the highest reaction rate constant (*k*) was 0.0116 min^−1^, confirming SrTiO_3_‐TiO_2_ heterojunction had the best degradation efficiency for TCH under sonopiezo‐catalysis. This is because the magnetic stirrer created the rotational field to the fluid medium, which can induce the stress on the sonocatalysts and further improve the piezoelectric response. Meanwhile, magnetic stirrer also promoted formation of vortex and the made the oxygen enter the solution, accompanied the effect of sonication‐induced mass transfer [63]. Because the ultrasonic agitation induces cavitation, microstreaming, and acoustic streaming in the reaction mixture, resulting in the formation of smaller and more uniform NPs with increased surface area [64]. Thus, both factors will effectively improve the synergistic efficiency between sonocatalysis and piezocatalysis on the removal of TCH.

In addition, the performance of SrTiO_3_‐TiO_2_ heterojunction for TCH degradation under various concentrations, pH and ultrasonic power was investigated. The initial pH value of TCH was set as 5 and the experiment was conducted under the condition of 300 W ultrasound power. Meanwhile, the degradation efficiency of TCH at various concentrations (10, 20, 30, and 40 mg L^−1^) of SrTiO_3_‐TiO_2_ heterojunction were 94.1%, 89.2%, 68.7%, and 55.7%, respectively (Figure S5). This phenomenon may be because high pollutant concentration obstructed the transport path of carriers and hindered the migration of carriers, weakening the sonopiezo‐catalytic performance [65]. Additionally, a significant quantity of intermediates will be generated throughout the degradation process, which competed with TCH molecules for limited sonopiezo‐catalytic active sites, resulting in a decrease in sonopiezo‐catalytic activity [66, 67]. Moreover, SrTiO_3_‐TiO_2_ heterojunction exhibited a strong resistance to changes in environmental pH levels, and the degradation efficiency of TCH was not significantly affected in the pH range of 5–9 (Figure S6). When the pH dropped to 3, the increase of electrostatic repulsion lead to the decrease of adsorption performance. Figure S7 demonstrates a clear correlation between the increase in ultrasonic power and the corresponding improvement in catalytic performance. Obviously, comparing with the condition of 240 and 300 W, the removal rate reached 99.1% under 360 W. With the power increased, external stress acting on SrTiO_3_‐TiO_2_ heterojunction by ultrasound increased, resulting in higher piezoelectric deformation and performance [60]. Therefore, SrTiO_3_‐TiO_2_ heterojunction displayed high potential for application in the sonopiezo‐catalytic degradation of TCH.

Then, for the sake of the evaluation of the practical application prospect of SrTiO_3_‐TiO_2_ heterojunction/US system, the interference resistance and stability of this system, as well as its applicability to various pollutant degradation were investigated. As shown in Figure 4E, with the concentration of Cl^−^ and SO_4_^2−^ increased, the degradation efficiency of TCH all display a gradual downward trend. It indicates that the Cl^−^ and SO_4_^2−^ might convert strong oxidative radicals into ROS with lower oxidation capacity [68]. Additionally, with the concentration of HA increased, the degradation efficiency of TCH was decreased from 90.6% (none) to 77.3% (100 mg L^−1^), demonstrating that the SrTiO_3_‐TiO_2_ heterojunction exhibits a certain ability to resist interference of organic matter (Figure S8). Meanwhile, the competition between inorganic anions and TCH may also contribute to a decline in degradation efficiency [69]. In addition, the cycle experiment was performed on the SrTiO_3_‐TiO_2_ heterojunction to evaluate its reusability and stability. After four consecutive rounds of 3 h continuous vibration, the degradation of TCH dropped from 93.1% to 81.2%, suggesting that SrTiO_3_‐TiO_2_ heterojunction exhibited excellent sonopiezo‐catalytic performance and stability (Figure 4F). The XRD and XPS of fresh and used SrTiO_3_‐TiO_2_ heterojunction was also tested to verify the stability of the SrTiO_3_‐TiO_2_ heterojunction. By comparison, the position and numbers of diffraction peak did not change in the XRD pattern of the used SrTiO_3_‐TiO_2_ heterojunction, suggesting the stability of SrTiO_3_‐TiO_2_ heterojunction (Figure S9). After the TCH degradation reaction, the XPS spectra of Ti 2p and Sr 3d of the used SrTiO_3_‐TiO_2_ heterojunction have no valence change and the positions of corresponding peak in Ti 2p and Sr 3d have not shifted (Figure S10). Additionally, the HRTEM of the used SrTiO_3_‐TiO_2_ heterojunction is shown in Figure S11. The corresponding lattice fringe images and corresponding FFT patterns graph demonstrate that the used SrTiO_3_‐TiO_2_ heterojunction still exist (110) crystal planes of SrTiO_3_ and (101) crystal planes of TiO_2_, suggesting that there is no obvious change in its morphological and structure (Figure S11B,C). Finally, ICP was used to determine that leaching percentage of Ti and Sr in the SrTiO_3_‐TiO_2_ heterojunction was approximately 0.1% and 0.2%, respectively. These results demonstrate that the SrTiO_3_‐TiO_2_ heterojunction has kind stability.

Thus, to better reveal the applicability to various antibiotic contaminants degradation in the SrTiO_3_‐TiO_2_ heterojunction system, the CIP, NOR, AMO, TCH, and AZM are selected as typical antibiotic pollutants and these antibiotic structures are classified by the corresponding functional groups. The electron‐deficient groups are marked in blue and the electron donating groups were marked in red (Figure 4G). The AMO, TCH, and AZM with no halogen group have a large amount of electron donating groups (─OH, ─NH_2_ etc.). NOR and CIP have the similar structural formulas, containing three strong electron donating groups and electron‐deficient halogenated groups (─F) and the cyclopropyl of CIP has a stronger ability to supply electron than ─CH_3_ of NOR. Additionally, it has reported that the ionization potential (IP) of organic compounds may affect the reaction rates [70]. Thus, the IP value of various antibiotic was calculated according to the reported density functional theory method shown in Table S1. For antibiotics containing halogenated groups, NOR (7.72 eV) has a higher IP value than CIP (7.43 eV). As for AMO, TCH and AZM containing non‐halogenated groups, the IP are calculated to be 8.22 eV, 8.08 eV, and 7.42 eV, respectively. As shown in Figure 4H, the SrTiO_3_‐TiO_2_ heterojunction shows an excellent degradation effect on TCH, AMO, and AZM but exhibited a poor degradation efficiency on NOR and CIP, which can attribute to selective degradation of electron rich pollutants by ^1^O_2_ [71]. Meanwhile, for halogenated antibiotics, the cyclopropyl of CIP with lower IP value have a stronger ability to supply electron than ─CH_3_ of NOR, contributing to the better removal rate than NOR and AMZ with the lowest IP value and the almost non‐halogenated antibiotics with electron donating groups resulting in the excellent removal rate [71, 72]. These results indicated that the selective ROS such as ^1^O_2_ may be generate in the SrTiO_3_‐TiO_2_ heterojunction system and play a key role in the reaction process.

### 3.3 Mechanism of Sonopiezo‐Catalysis

**FIGURE 5:**
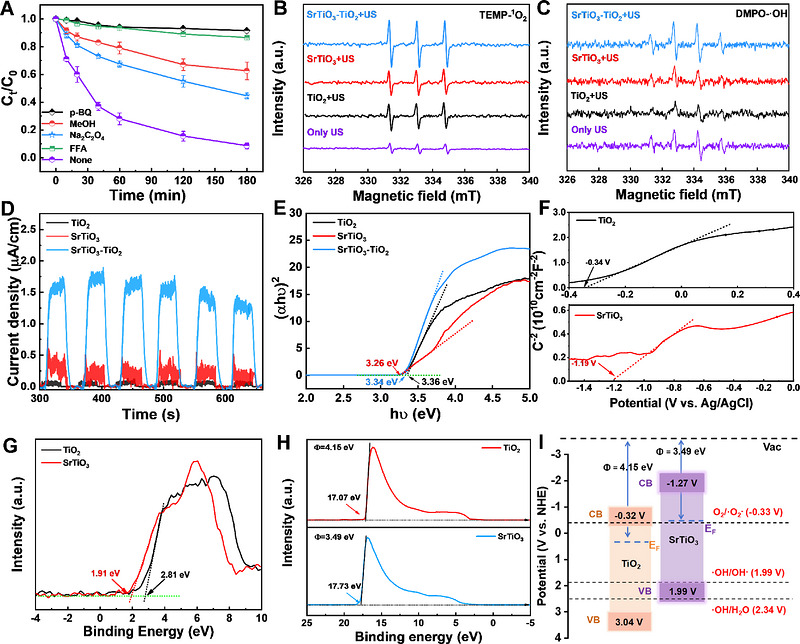
(A) Free radical scavenging experiments of SrTiO_3_‐TiO_2_ heterojunction for TCH degradation under ultrasonic vibration. EPR spectra of (B) ^1^O_2_ and (C) •OH formed in different sonopiezo‐catalytic systems. (D) EIS Nyquist plots, (E) Transient sonopiezo‐current of TiO_2_ NRs, SrTiO_3_ NPs, and SrTiO_3_‐TiO_2_ heterojunction. (F) Band gap spectra, (G) MS curves, and (H) VB‐XPS of TiO_2_ NRs, and SrTiO_3_ NPs. (I) the possible energy band structure diagram of the SrTiO_3_‐TiO_2_ heterojunction.

To insight into the coupled mechanism of sonopiezo‐catalytic process, charge transport, band structure, and the identification of ROS were systematically investigated. The radical quenching experiments were carried out with MeOH, p‐BQ, FFA, and Na_2_C_2_O_4_ used as the radical quenching agents to determine the main ROS during the degradation process of TCH. As presented in Figure 5A, the removal rate of TCH was declined to 33.2% with the addition of MeOH, suggesting •OH was not the main active species to TCH degradation. Additionally, the •O_2_^−^ is also a significant ROS reported to be produced during degradation of various pollutants, and the contribution to TCH degradation was assessed by introducing p‐BQ to the system. After the addition of p‐BQ, the degradation and reaction rate of TCH were effectively suppressed by comprising the *k* constant change (Figure S12). Thus, •O_2_^−^ was certified to make great contribution to TCH degradation. Furthermore, previous study has reported that •O_2_^−^ could be oxidized by h^+^ to participate in the formation of ^1^O_2_ rather than directly involved in the degradation reaction, due to the low redox potential of O_2_/•O_2_^−^ (−0.33 eV) [71]. To identify that ^1^O_2_ could be generated from the recombination of •O_2_^−^, FFA was used to be an effective scavenger of ^1^O_2_ in scavenging experiment [73]. Result showed that the clear suppression of TCH degradation efficiency was carried out in the presence of FFA, indicating that ^1^O_2_ was the dominant reactive species in the degradation reaction. Moreover, EPR experiments were employed to detect the generation of •OH and ^1^O_2_, using DMPO and TEMP as their spin‐trapping agents, respectively [71]. Figure 5B,C clearly demonstrated the existence of •OH and ^1^O_2_ in the all systems. In the only US system, the H_2_O is decomposed into •OH and •H for the reason of cavitation effect [2], and •OH is converted to the •OOH intermediate by reacting with O_2_, which is finally decomposed into ^1^O_2_ [74]. Meanwhile, the addition of heterogeneous catalysts effectively enhances the signal intensity of ROS. Notably, the EPR signal of ^1^O_2_ and •OH in SrTiO_3_‐TiO_2_ heterojunction can observe the strongest intensities, comparing with the TiO_2_ NRs, SrTiO_3_ NPs, and only US, suggesting an improvement in generation of ^1^O_2_ and •OH for the formation of the heterojunction. Thus, the quenching experiment and EPR test jointly proved that •OH, ^1^O_2_, •O_2_^−^, and h^+^ all participated in the sonopiezo‐catalytic process for TCH degradation over SrTiO_3_‐TiO_2_ heterojunction and the •O_2_^−^ was oxidized by h^+^ and further converted to ^1^O_2_.

The piezocatalytic characteristics primarily relies on the surface charge produced by internal charge transfer, which was driven by the piezoelectric potential [75]. Consequently, we propose to investigate the efficiency of charge transfer after the construction of a SrTiO_3_‐TiO_2_ heterojunction by electrochemical experiment. The equivalent circuit was shown in the inset of Figure S13 and the intercept in the high frequency represented the equivalent series resistance (Rs), the high frequency semicircle represented the charge transfer resistance (Rct), while there was a diffusion resistance in the mid‐frequency region (W), and Cd represented the corresponding capacitance. In contrast to TiO_2_ NRs and SrTiO_3_ NPs, the SrTiO_3_‐TiO_2_ heterojunction exhibited a significantly smaller arc radius in EIS Nyquist plots, suggesting low transfer resistance inside SrTiO_3_‐TiO_2_ heterojunction to facilitate interface charge transfer [76]. The above electrochemical results reflected the improvement of sonocatalytic activity was mainly ascribe to accelerated transfer behavior of charge carriers via formation of heterojunction [52, 77]. Further study on the carrier transfer behavior was carried out by transient piezoelectric current response experiments (Figure 5D). During test, the three catalysts were subjected to periodic switching of ultrasonication (switching every 30 s) to simulate transient conditions. The SrTiO_3_‐TiO_2_ heterojunction presents a high sonopiezo‐current of about 1.5 µA cm^−1^, which is obviously stranger than that of TiO_2_ NRs (∼0.07 µA cm^−1^) and SrTiO_3_ NPs (∼0.45 µA cm^−1^), highlighting that a high catalytic activity is achieved through increased charge separation which is facilitated by the construction of SrTiO_3_‐TiO_2_ heterojunction [78].

To further investigate the electron transfer mechanism of the SrTiO_3_‐TiO_2_ heterojunction induced by ultrasound, the energy band structure of TiO_2_ NRs and SrTiO_3_ NPs were measured by the MS and UV‐DRS tests (Figure S14). Comparing with TiO_2_ NRs, the UV absorption capacity of SrTiO_3_‐TiO_2_ heterojunction had been significantly improved. According to Kubelka–Munk's formula, A(*hν*−*Eg*) = (*αhν*)^1/n^ (*α*, *h*, *ν*, *A*, and *Eg* represent absorption coefficient, Planck constant, optical frequency, constant, and band gap energy, respectively) [22], the *Eg* of TiO_2_ NRs and SrTiO_3_ NPs and SrTiO_3_‐TiO_2_ heterojunction were calculated to be 3.36, 3.34, and 3.26 eV, respectively (Figure 5E). Figure 5F displayed the corresponding flat‐band potentials of TiO_2_ NRs (−0.34 V) and SrTiO_3_ NPs (−1.19 V) (vs. Ag/AgCl). And the final SrTiO_3_‐TiO_2_ heterojunction exhibits a low flat‐band potential of −0.48 V than that of pure TiO_2_, which reveals that the introduction of SrTiO_3_ produces a lower flat‐band potential (Figure S15). Additionally, TiO_2_ NRs and SrTiO_3_ NPs exhibited positive slopes on their MS curves, indicating TiO_2_ NRs and SrTiO_3_ NPs were n‐type semiconductor [22]. In general, *E*_CB_ was approximately 0.20 V more positive than flat‐band potentials in the n‐type semiconductor [22, 79]. Thus, the *E*_CB_ values of TiO_2_ NRs and SrTiO_3_ NPs were determined to be −0.54 V and −1.39 V (vs. Ag/AgCl), respectively. Based on the conversion formula: *E*_NHE_ = *E*_Ag/AgCl_ + 0.197 V [80], the corresponding *E*_CB_ of TiO_2_ NRs and SrTiO_3_ NPs relative to the normal hydrogen electrode (NHE) (pH = 7)was determined to be −0.34, and −1.19 eV (vs. NHE), respectively. Meanwhile, the valence band‐XPS (VB‐XPS) spectra was employed to investigate the intrinsic electronic structure of TiO_2_ NRs and SrTiO_3_ NPs (Figure 5G). The valence band maximum (*E*_VBM_) relative to the NHE (pH = 7) of TiO_2_ NRs and SrTiO_3_ NPs were subsequently calculated by (Equation 2): [81]

(2) $$E_{\mathrm{VBM}(\mathrm{NHE})}=\varphi +E_{\mathrm{VBM}(\mathrm{VB}-\mathrm{XPS})}-4.44$$

where *φ* is the electron work function of the analyzer (4.6 eV), *E*_VBM (VB‐XPS)_ is obtained from VB‐XPS spectra. Accordingly, the *E*_VBM_ values of TiO_2_ and SrTiO_3_ were 2.81 eV and 1.91 eV (vs. NHE), respectively, which could be further demonstrated by the empirical formula *E*_VB_ = *E*_CB_ + *E*_g_ [82]. In general, the work function of a material is negatively related to its Fermi level. To further determine the charge transfer direction at the heterojunction interface of SrTiO_3_‐TiO_2_, the work function (*Φ*) of TiO_2_ and SrTiO_3_ was evaluated by ultraviolet photoelectron spectroscopy (UPS). As shown in Figure 5H, the cut‐off energy values of TiO_2_ and SrTiO_3_ are 17.08 eV and 17.73 eV, respectively. The work functions of TiO_2_ and SrTiO_3_ can be determined to be 4.15 and 3.49 eV by subtracting the He I excitation energy (21.22 eV), respectively [83]. Results suggest that SrTiO_3_ possesses a higher Fermi level, due to its lower work function than that of TiO_2_. Thus, electrons in SrTiO_3_ spontaneously can flow to TiO_2_, causing a balanced Fermi energy levels and generating an internal electric field at the interface of SrTiO_3_‐TiO_2_ heterojunction [83]. Above all, the band structure diagram of TiO_2_ and SrTiO_3_ could be obtained as shown in Figure 5I. According to the change of electron density in XPS spectra and staggered band structure between TiO_2_ and SrTiO_3_, the e^−^ likely transferred from SrTiO_3_ to TiO_2_ in the SrTiO_3_‐TiO_2_ heterojunction. During the catalytic process, electron transport mode can only generate S‐scheme or type‐II heterojunction interfaces under the US radiation [26]. Contrary to the results of the scavenging experiment, the type‐II heterojunction was unable to overcome the potential barrier of O_2_/•O_2_^−^ (−0.33 eV) for SrTiO_3_‐TiO_2_ heterojunction effectively [26]. Thus, the transfer pathway of charge in SrTO_3_‐TiO_2_ heterojunction was more consistent with features of S‐scheme heterojunction.

**SCHEME 2:**
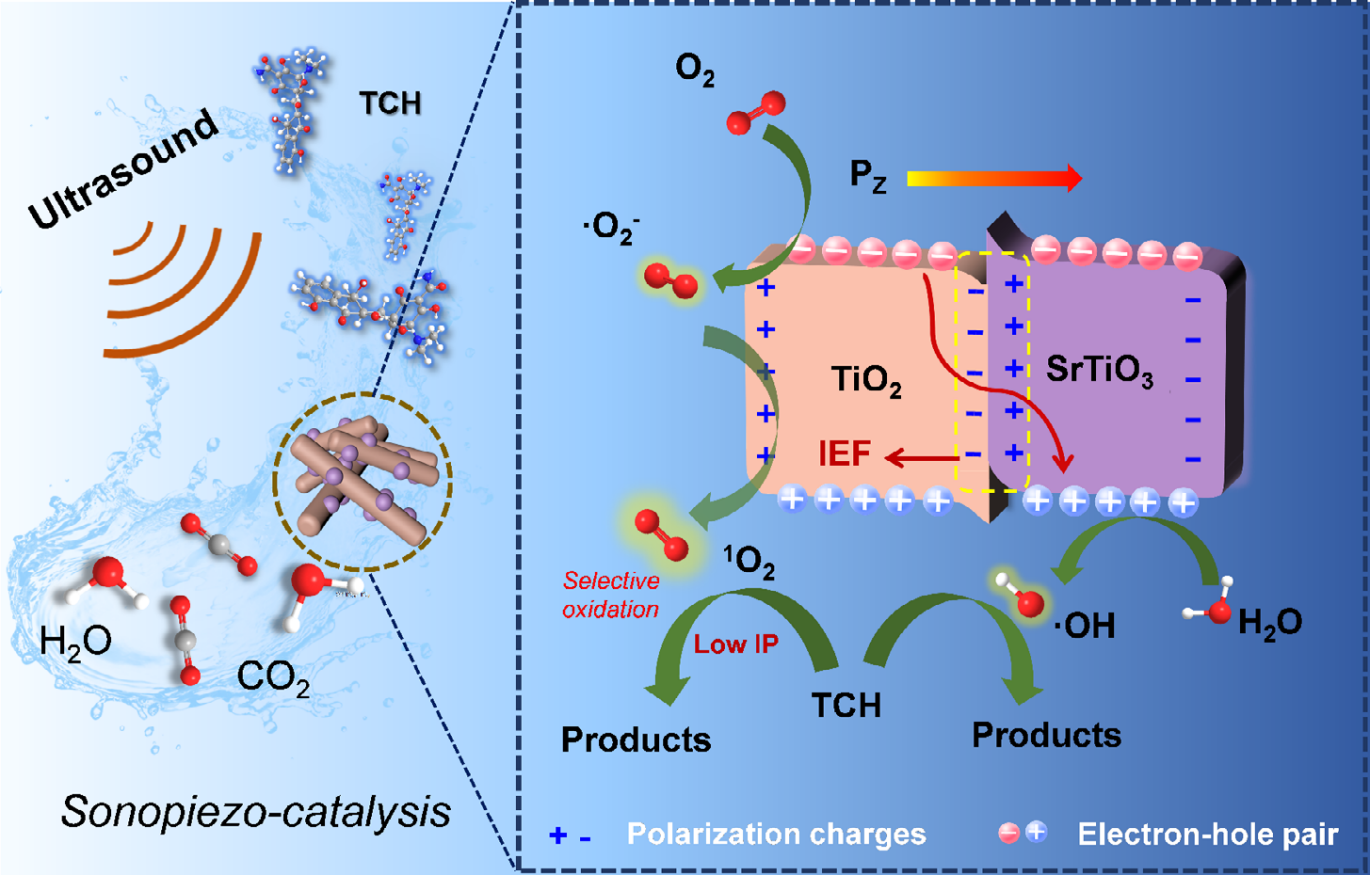
Illustration of sonopiezo‐catalytic mechanism of SrTiO_3_‐TiO_2_ heterojunction.

Finally, based on above characterization and experiment results, the possible sonopiezo‐catalytic mechanism of SrTiO_3_‐TiO_2_ S‐scheme heterojunction was showed in Scheme 2. Under sonopiezo initiation, the CB electrons of the SrTiO_3_ NPs was excited and spontaneously transferred towards the CB surface of TiO_2_ across their interface, resulting in the band bending of TiO_2_ and SrTiO_3_ due to the consumption and accumulation of electrons [26]. Then, the semiconductor‐piezoelectric interaction between TiO_2_ and SrTiO_3_ creates a strong IEF across the interface and modulates the charge distribution. And the formed IEF regulates the electron flow and contributes to the charge transfer due to its rectifying properties. Therefore, the charge separation between VB and CB of SrTiO_3_‐TiO_2_ heterojunction could be carried out under US irradiation (Equation 3). The sonopiezo‐generated e^−^ can be captured by O_2_ in the solution, producing •O_2_^−^ (Equation 4) [52], while the separated h^+^ will react with water molecule to generate •OH (Equation 5) [47]. Dramatically, the formation of S‐scheme heterojunction in SrTiO_3_‐TiO_2_ heterojunction not only suppresses the recombination of e^−^–h^+^ pairs, but also promotes the formation piezoelectric polarization electric field (Pz). This result, in turn, destroys the interface shielding charge layer and ensure the continuous operation of the IEF [84], and it is potentially conducive to the regulation of ^1^O_2_ evolution by combination of •O_2_^−^ with sonopiezo‐generated h^+^ (Equation 6) [73], under ultrasonic mechanical stress. Abundant ^1^O_2_ active species creates the efficient and selective degradation capacity of various antibiotic contaminations containing electron‐rich moieties, including TCH, AZM, and AMO. While the generated •OH only leads to the partial oxidation degradation of antibiotic contaminations, producing CO_2_ and H_2_O.

(3) CatalysisUS→Catalysis*(e−+h+)

(4) $$O_{2}+e^{-}\to •O_{2}^{-}$$

(5) $$H_{2}O+h^{+}\to •\mathrm{OH}+H^{+}$$

(6)

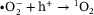




## 4 Conclusion

In summary, SrTiO_3_‐TiO_2_ heterojunction was successfully synthesized to triggered ^1^O_2_ evolution system for the selective removal of TCH. Meanwhile, we discussed the performance of SrTiO_3_‐TiO_2_ heterojunction and underlying mechanisms of catalytic efficacy in sonopiezo‐catalytic for the effective mineralization of TCH by SrTiO_3_‐TiO_2_ heterojunction. All results demonstrate that SrTiO_3_‐TiO_2_ heterojunction with a high d_33_ (43.5 pm V^−1^) and sonopiezo‐current (1.5 µA cm^−1^) exhibits an excellent sonopiezo‐catalytic performance. Moreover, the intrinsic activity of the SrTiO_3_‐TiO_2_ heterojunction is boosted for the role of IEF and piezoelectric electric field in interface of S‐scheme heterojunction, which enhances the separation and transfer efficiency of charge carrier during the sonopiezo‐catalytic TCH degradation reaction process. Moreover, we identified the underlying mechanism as continuous generation of ^1^O_2_, wherein sonopiezo‐generated e^−^ initiates the formation of •O_2_^−^, which is subsequently transformed into ^1^O_2_, leading to a higher selectivity in attacking the antibiotic contaminations containing electron‐rich moieties. This study is conducive to provide new insights into designing and developing sonopiezo‐catalytic system for selective oxidation of electron‐rich pollutants.

## Author Contributions

**Weiwei Wang**: conceptualization, writing – review & editing, project administration, funding acquisition, formal analysis. **Chun Lu**: writing – original draft, methodology, supervision, resources, visualization, formal analysis, data curation, conceptualization. **Xiaoxiao Liu**: methodology, formal analysis. **Wenlong Yang**: investigation, software. **Chenyao Hu**: methodology, formal analysis, **Jie Zhou**: resources, project administration. **Guangze Nie**: visualization, resources, project administration, funding acquisition.

## Conflicts of Interest

The authors declare no conflicts of interest.

## Supporting information




**Supporting File 1**: exp270174‐sup‐0001‐SuppMat.docx.

**Supporting File 2**: exp270174‐sup‐0002‐SuppMat.pptx.

## Data Availability

The main data supporting the results in this study are available within the paper and its Supporting Information. The other data that support the findings of this study are available from the corresponding author upon reasonable request.
