# Supplementary material for: Acceleration of Singlet Oxygen Evolution by Sonopiezoelectric Charge Transfer Over SrTiO3‐TiO2 Heterojunction for Selective Oxidation
Source: Exploration (Beijing). 2026 May 28;6(3):20250012. doi: 10.1002/EXP.20250012 (PMC13317800; doi:10.1002/EXP.20250012)
Supplement: Supplementary file 1 — Supporting File 1: exp270174‐sup‐0001‐SuppMat.docx. [file EXP2-6-20250012-s001.docx]

**Acceleration of Singlet Oxygen Evolution by Sonopiezoelectric Charge Transfer over SrTiO_3_-TiO_2_ Heterojunctions for Selective Removal of Tetracycline**

Weiwei Wang^1^, Chun Lu^1^, Xiaoxiao Liu^1^, Wenlong Yang^1^, Jie Zhou^2,^*，Chenyao Hu^3^, Xin Li^4^, and Guangze Nie^1,^*

^1^School of Environmental Science and Engineering, Nanjing Tech University, Nanjing 211816, Peoples R China

^2^School of Chemistry and Chemical Engineering, Nanjing University of Science and Technology, Nanjing, 210094, Peoples R China

^3^Advanced Analysis and Testing Center, Nanjing Forestry University, Nanjing 210037, Peoples R China

^4^Singapore Membrane Technology Centre, Nanyang Environment and Water Research Institute, Nanyang Technological University, Singapore, 637141, Singapore

^†^These authors contributed equally.

^*^Corresponding author: [gznie@njtech.edu.cn](mailto:gznie@njtech.edu.cn) (G. Nie); [fnzhoujie@njust.edu.cn](mailto:fnzhoujie@njust.edu.cn) (J. Zhou)


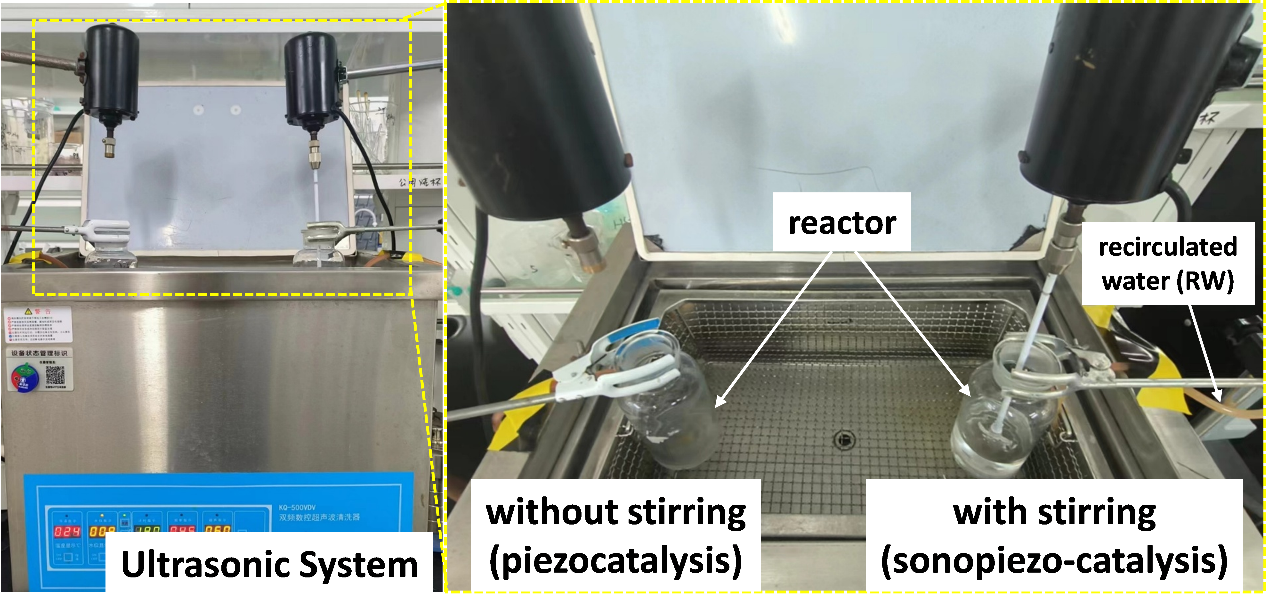


**Figure S1.** Experimental setup of the sono-piezocatalytic TCH degradation.


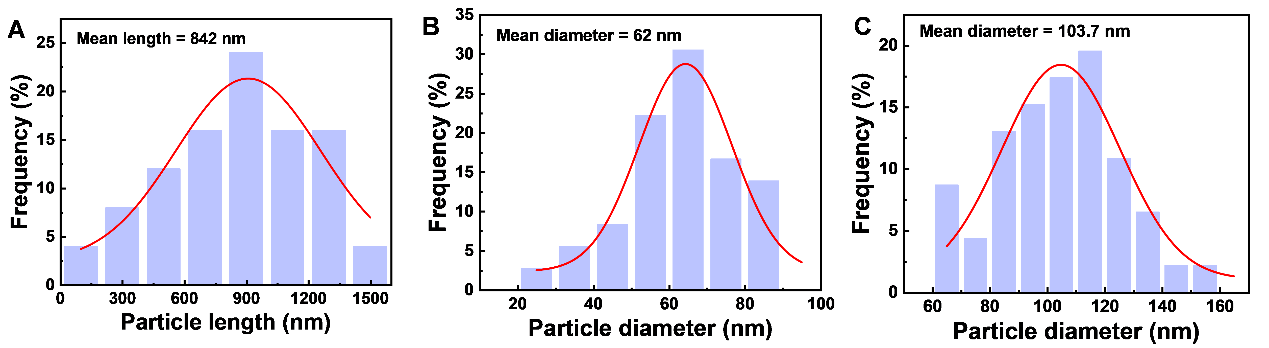


**Figure S2.** The frequency distribution of (a) length and diameter of (b) TiO_2_ NRs and (c) SrTiO_3_ NPs.


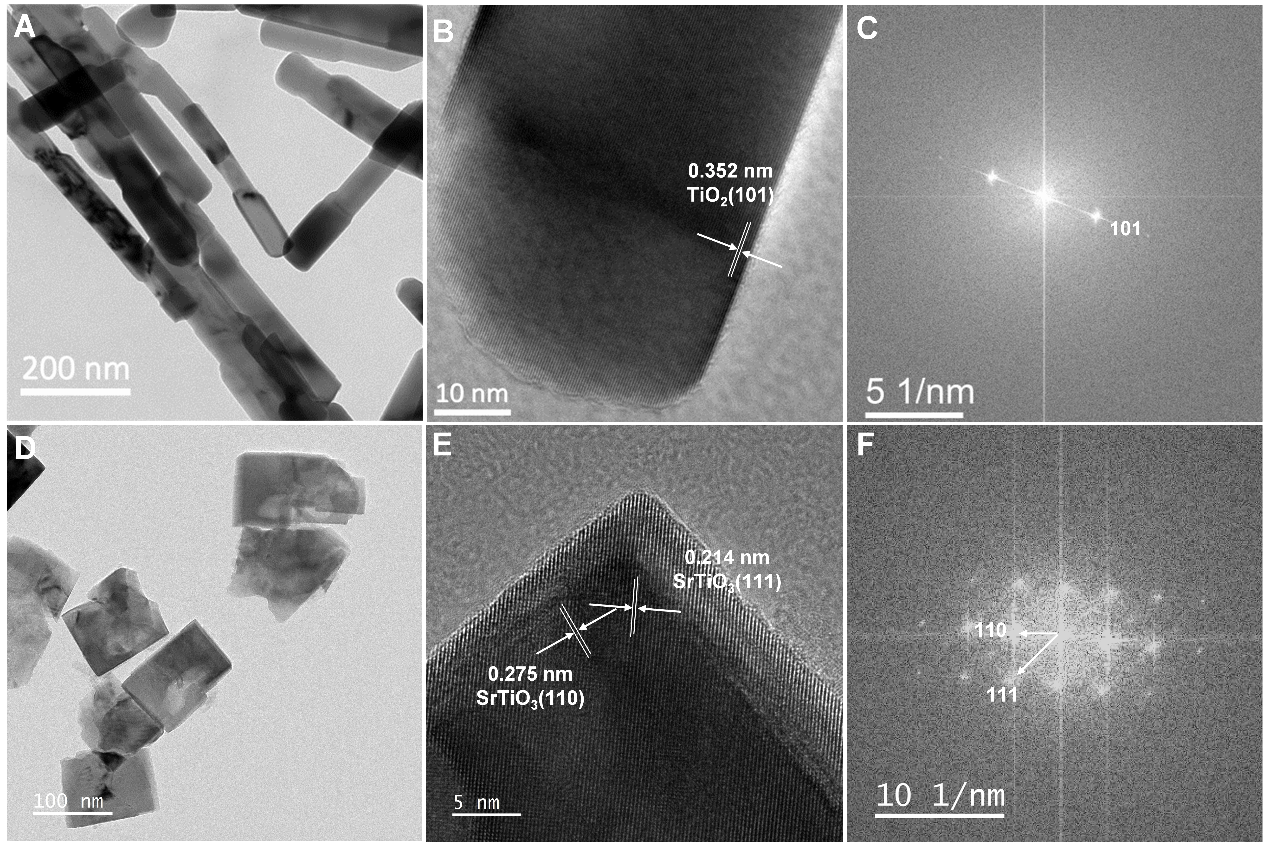


**Figure S3.** (A) TEM, (B) HRTEM, (C) Lattice fringe images for TiO_2_ NRs. (D) TEM, (E) HRTEM, (F) Lattice fringe images for SrTiO_3_ NPs.


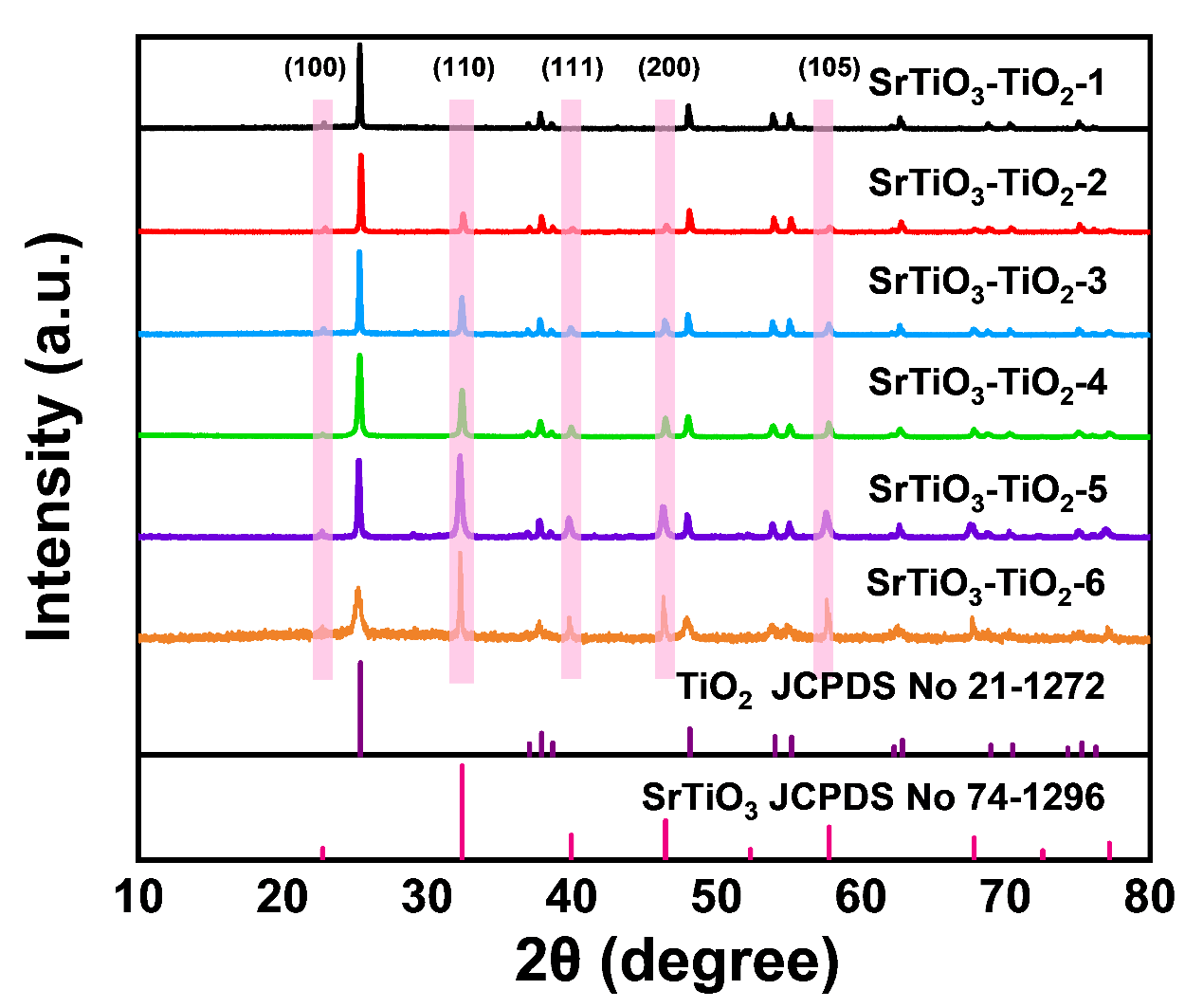


**Figure S4.** XRD patterns of SrTiO_3_-TiO_2_-X (X=1/2/3/4/5/6)


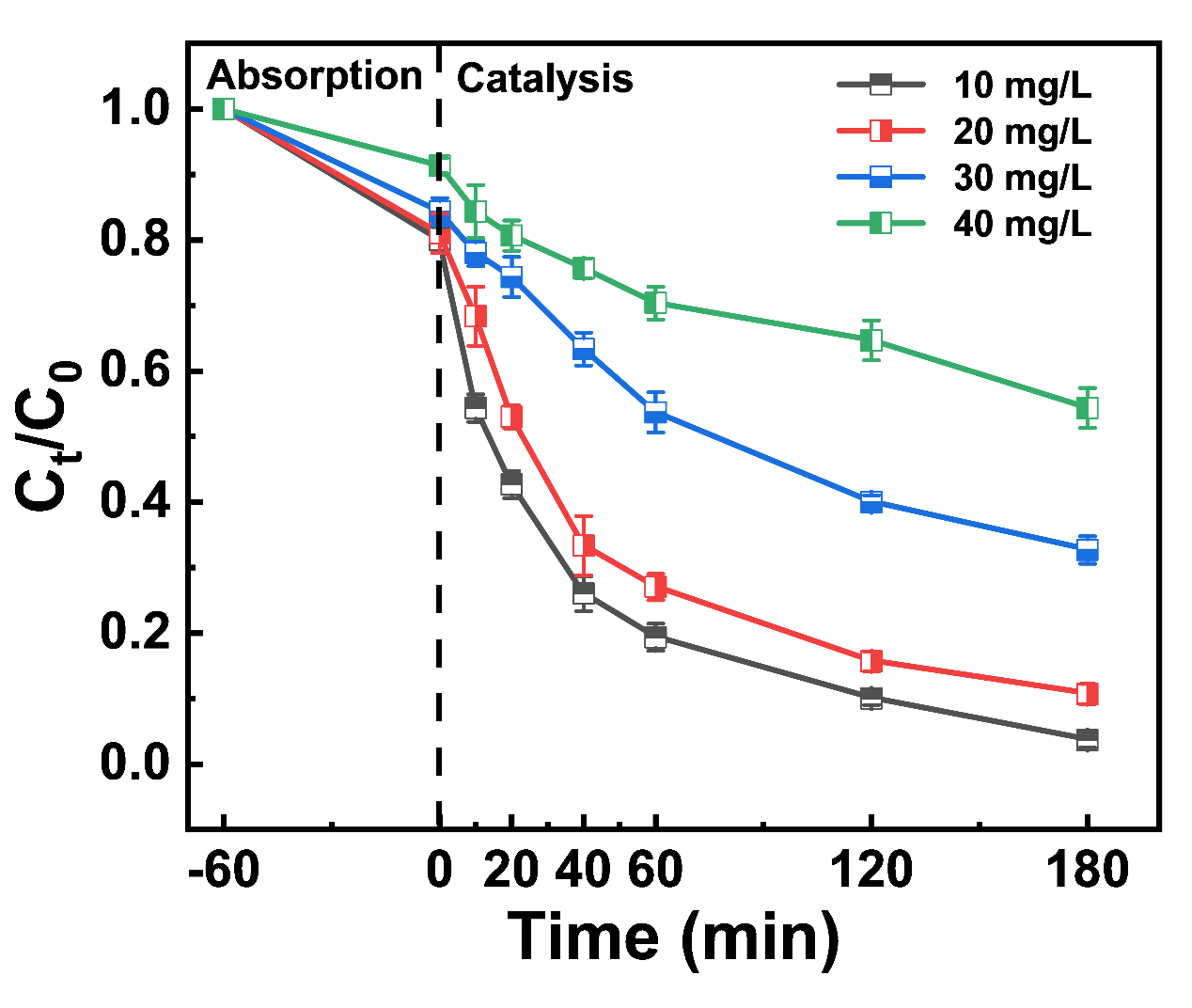


**Figure S5.** Effects of initial pollutant concentration on TCH degradation of SrTiO_3_-TiO_2_ heterojunction.

_
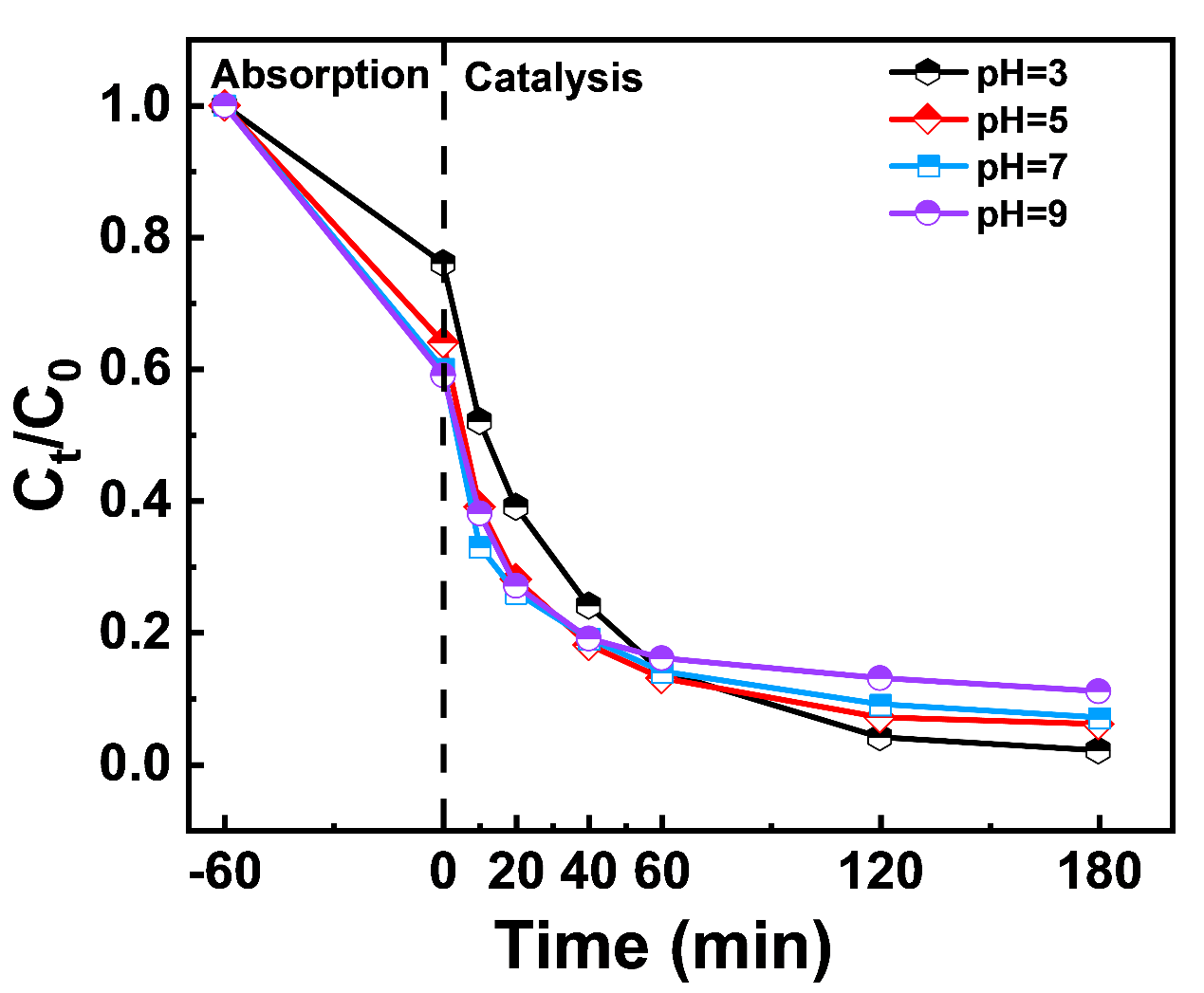
_**Figure S6.** Effects of initial solution pH on TCH degradation of SrTiO_3_-TiO_2_ heterojunction.

_
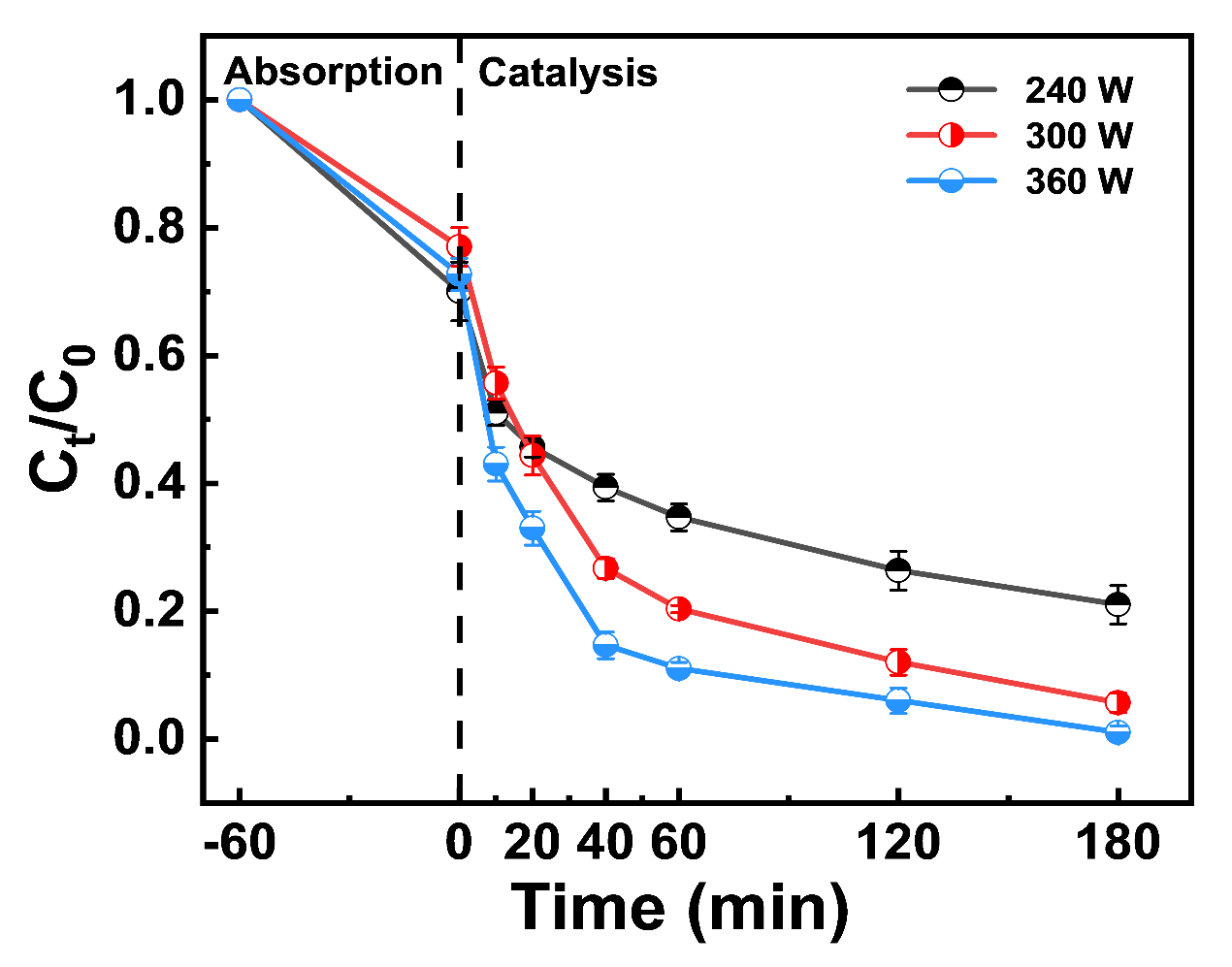
_**Figure S7.** Effects of ultrasonic power on TCH degradation of SrTiO_3_-TiO_2_ heterojunction.


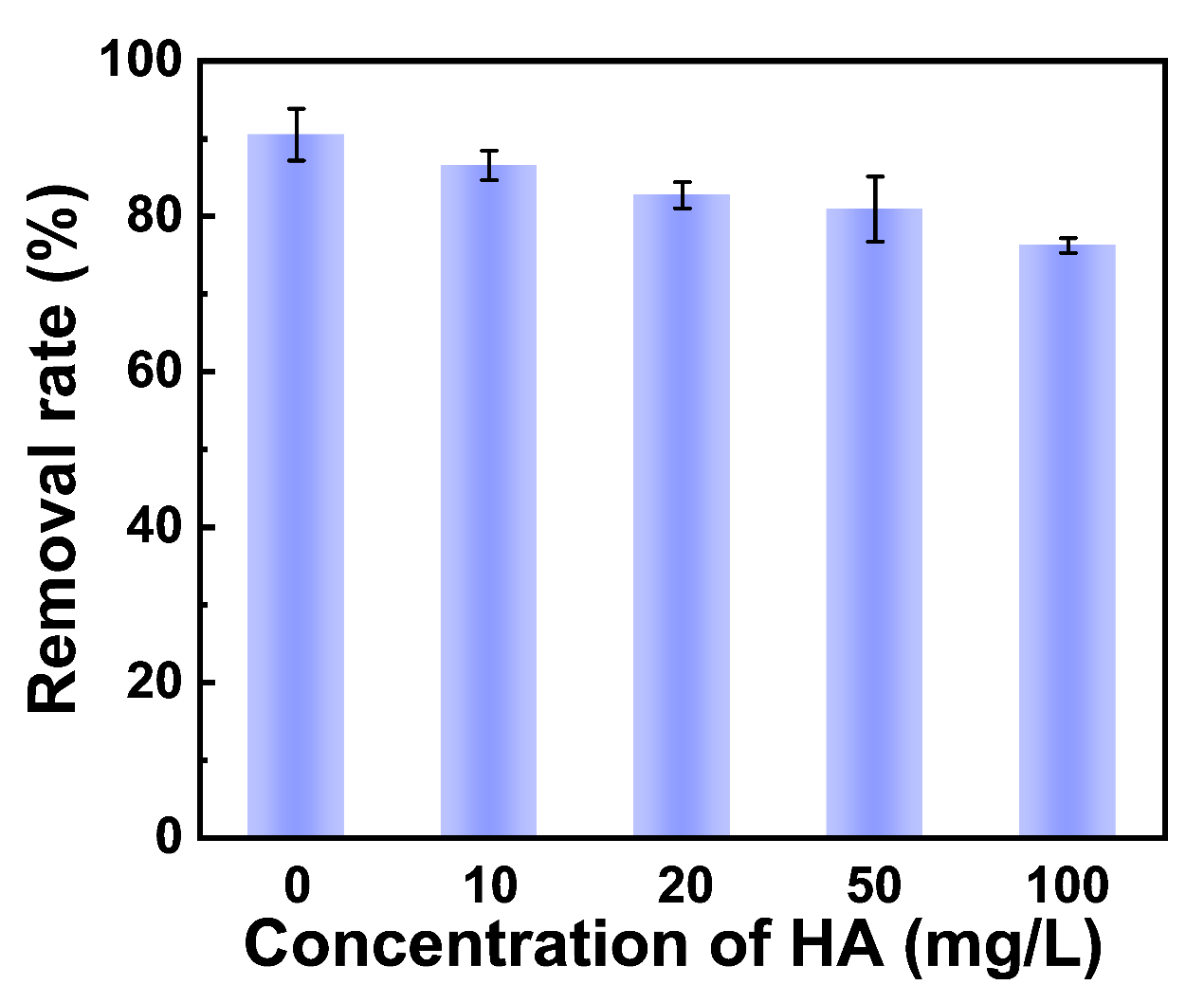


**Figure S8.** Effects of HA concentrations on TCH degradation by SrTiO_3_-TiO_2_ heterojunction


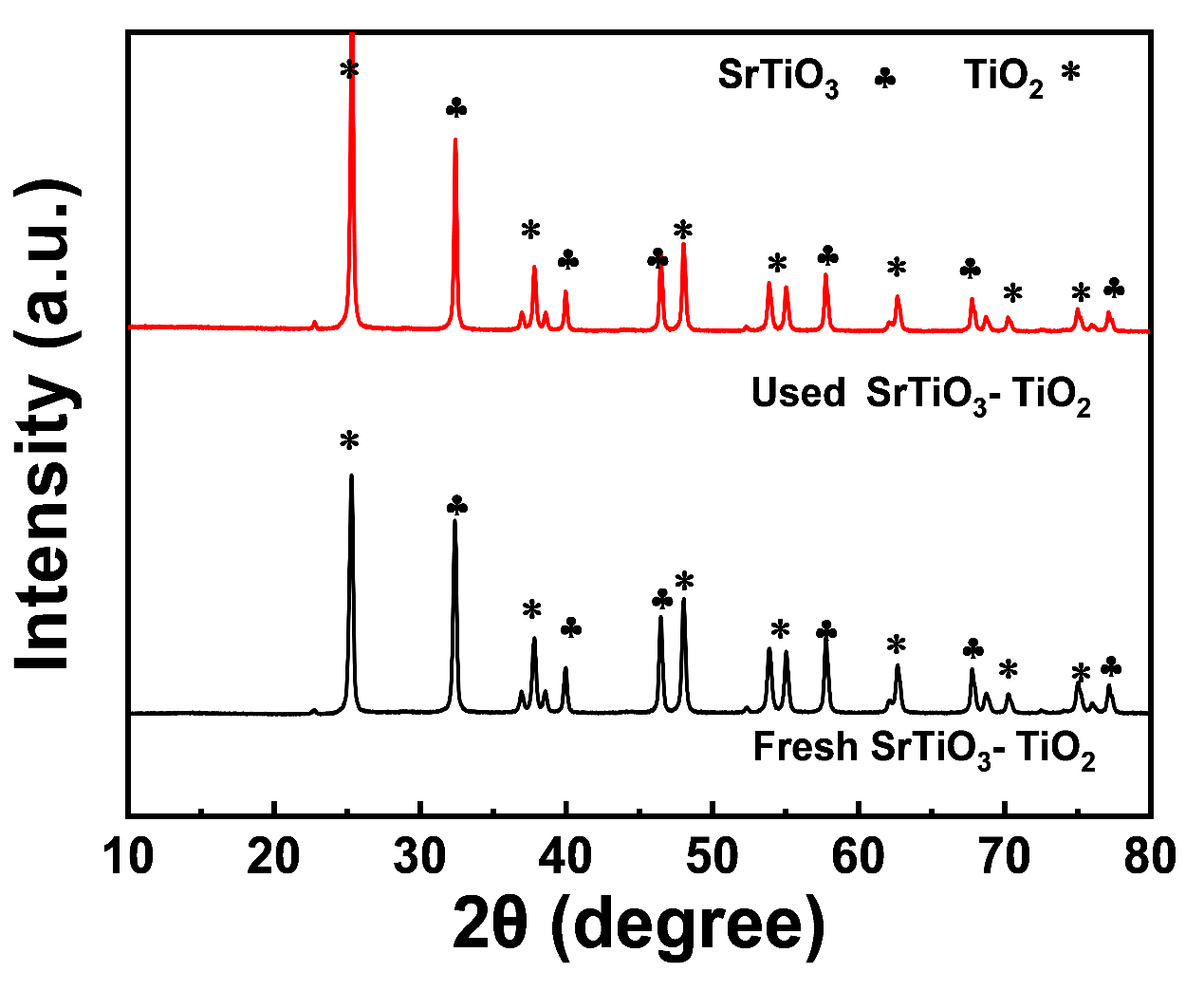


**Figure S9.** The comparison of PXRD pattern of fresh and used SrTiO_3_-TiO_2_ heterojunction.


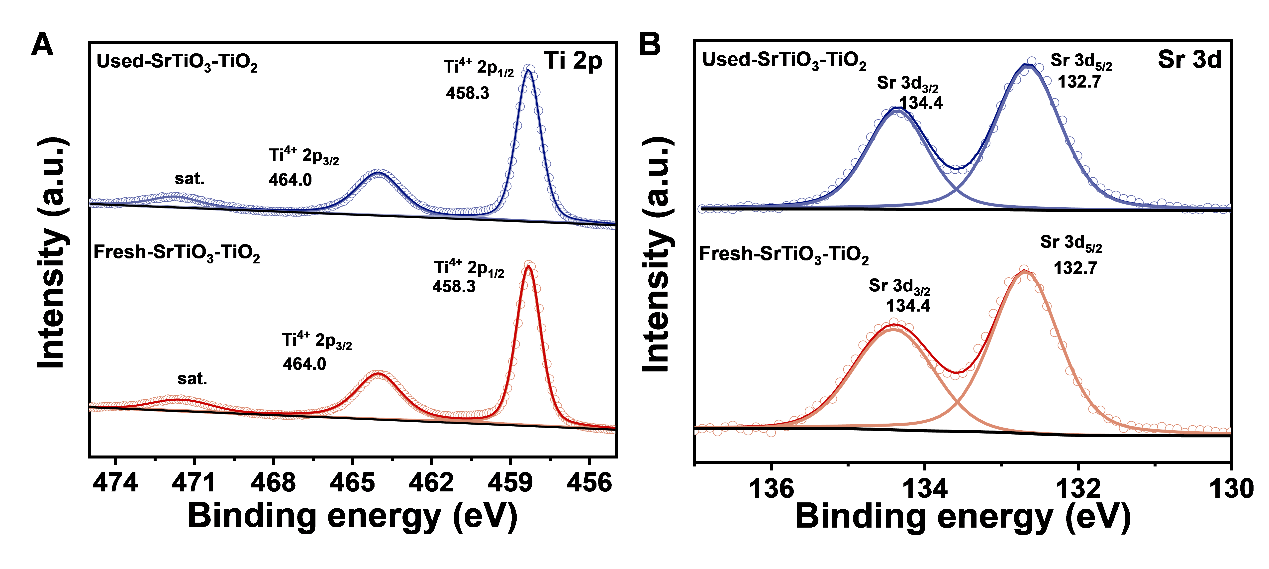


**Figure S10.** The comparison of XPS spectra of fresh and used SrTiO_3_-TiO_2_ heterojunction (A) Ti 2p, (B) Sr 3d.


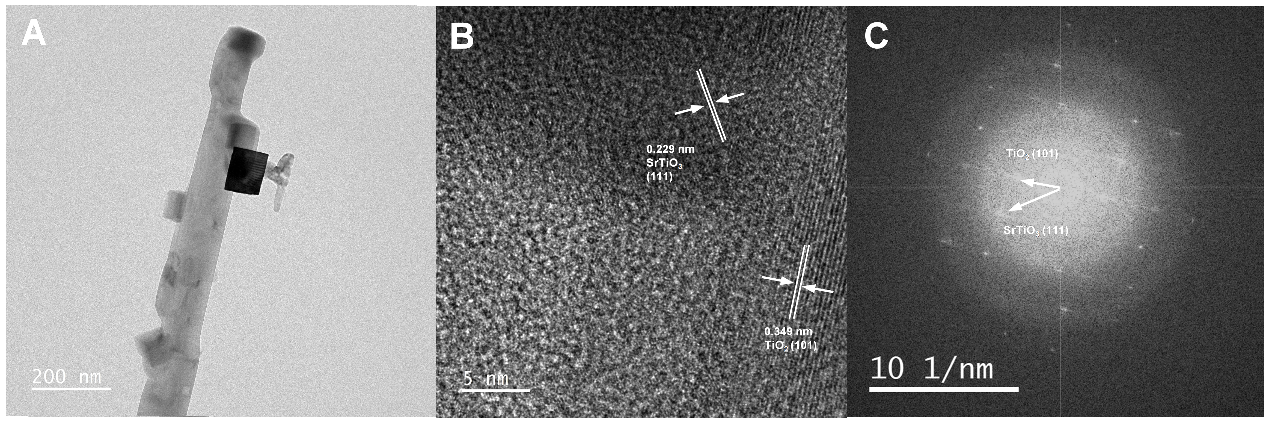


**Figure S11.** (a) TEM, (b) Lattice fringe images patterns, and (c) the corresponding FFT patterns of the used SrTiO_3_-TiO_2_ heterojunction.

**Table S1.** Structural formulas, ground state electronic energy, excited state electronic energy and ionization potentials (IPs) of various aromatic compounds.

| Antibiotic contaminant | Ground state electronic energy (E_G_, Hartree*) | Excited state electronic energy (E_E_, Hartree) | Ionization potentials (IP, eV) |
| --- | --- | --- | --- |
| AZM | -2502.5696 | -2502.2968 | 7.42 |
| TCH | -1563.49 | -1563.193 | 8.08 |
| AMO | -1559.2512 | -1558.9491 | 8.22 |
| NOR | -1109.886 | -1109.6023 | 7.72 |
| CIP | -1147.9519 | -1147.6788 | 7.43 |

*1 Hartree≈27.21 eV

All values were calculated according to the reported density functional theory method by using the M062X/6–31 G(df,p) theory level.^[1]^ The IP value is calculated by the following empirical formula: IP = E_G_ - E_E_.^[2]^

_
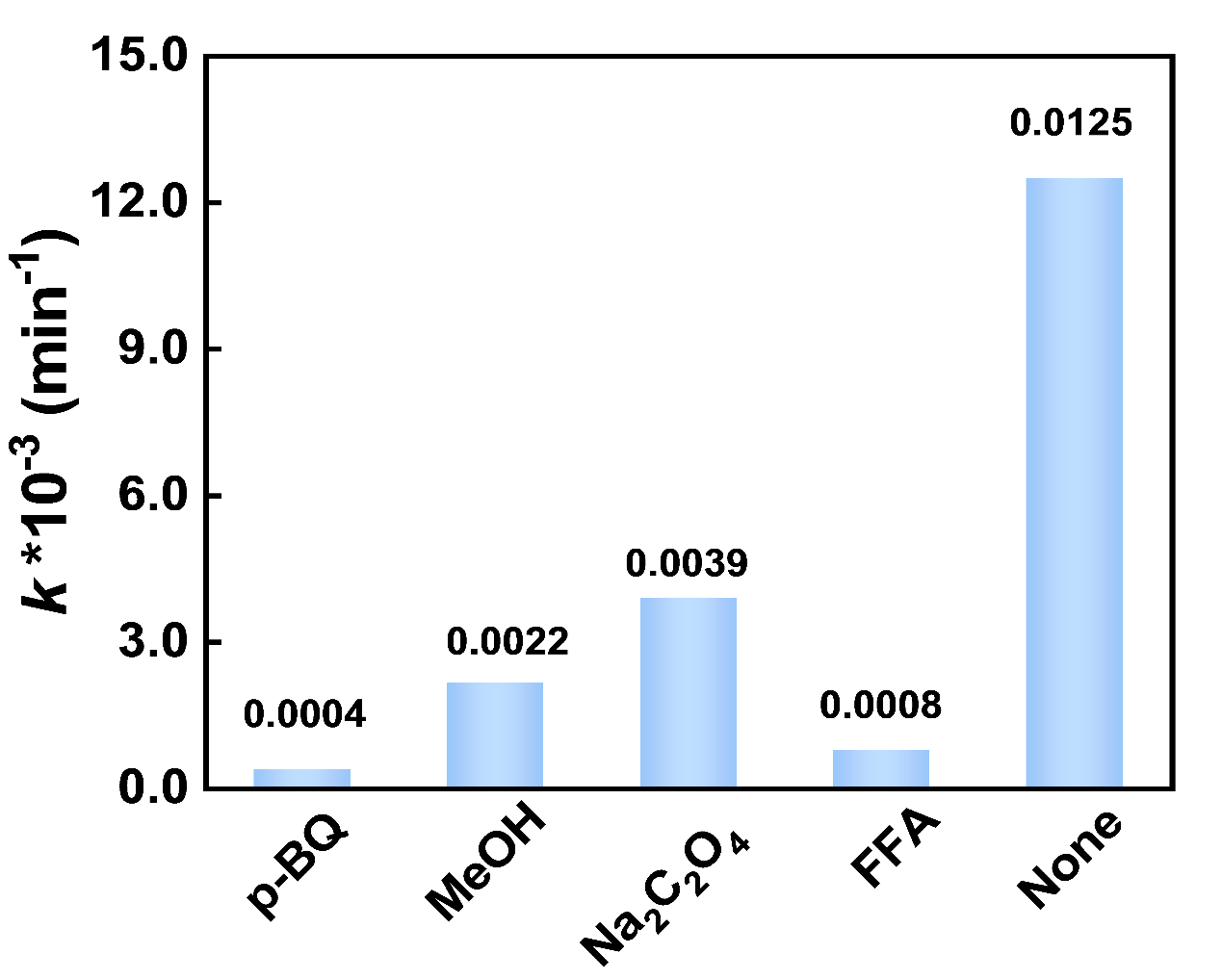
_

**Figure S12.** The reaction kinetic constant (k) of various scavengers for TCH degradation of SrTiO_3_-TiO_2_ heterojunction under ultrasound irradiation.

_
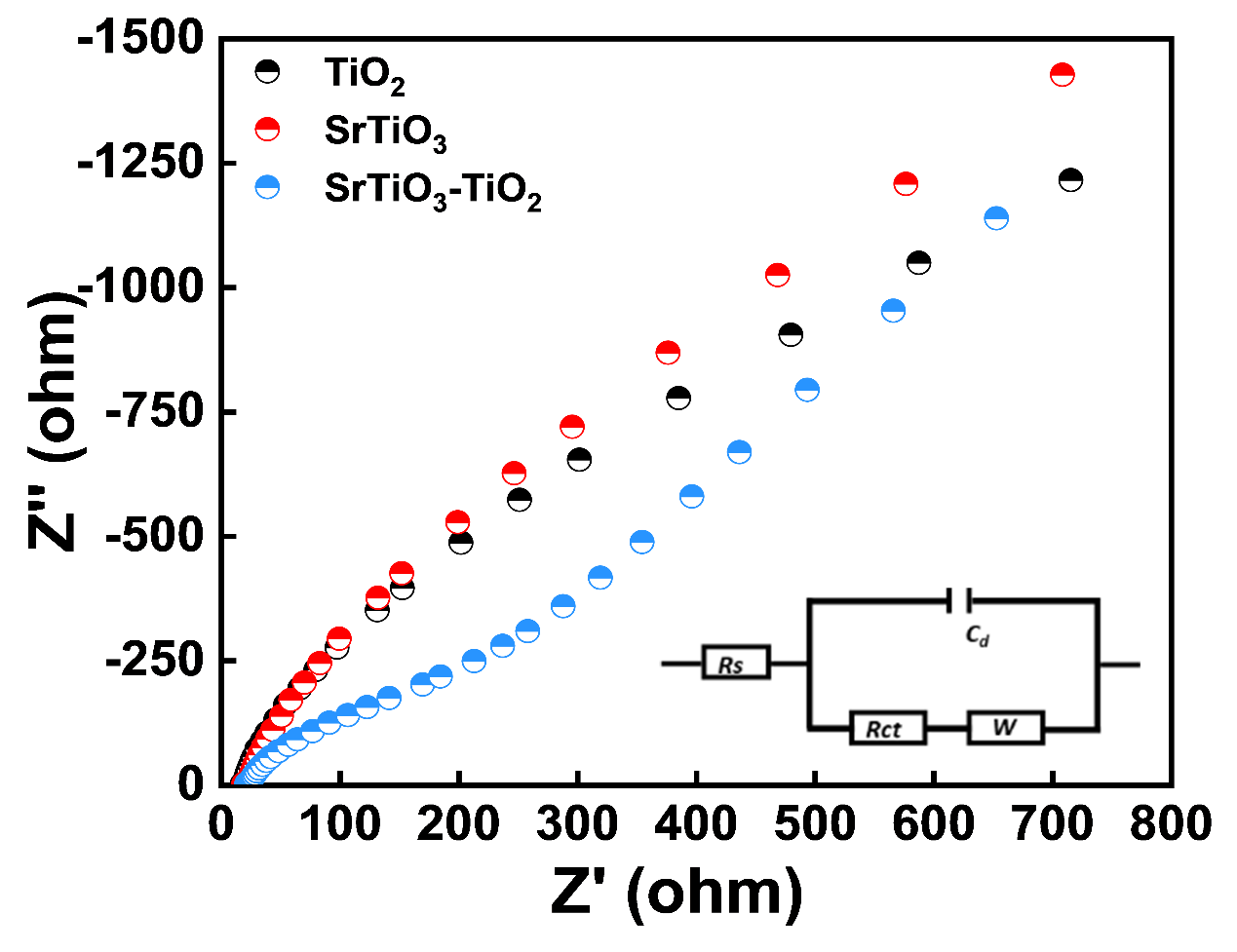
_

**Figure S13.** EIS Nyquist plots of TiO2 NRs, SrTiO3 NPs, and SrTiO3-TiO2 heterojunction.

_
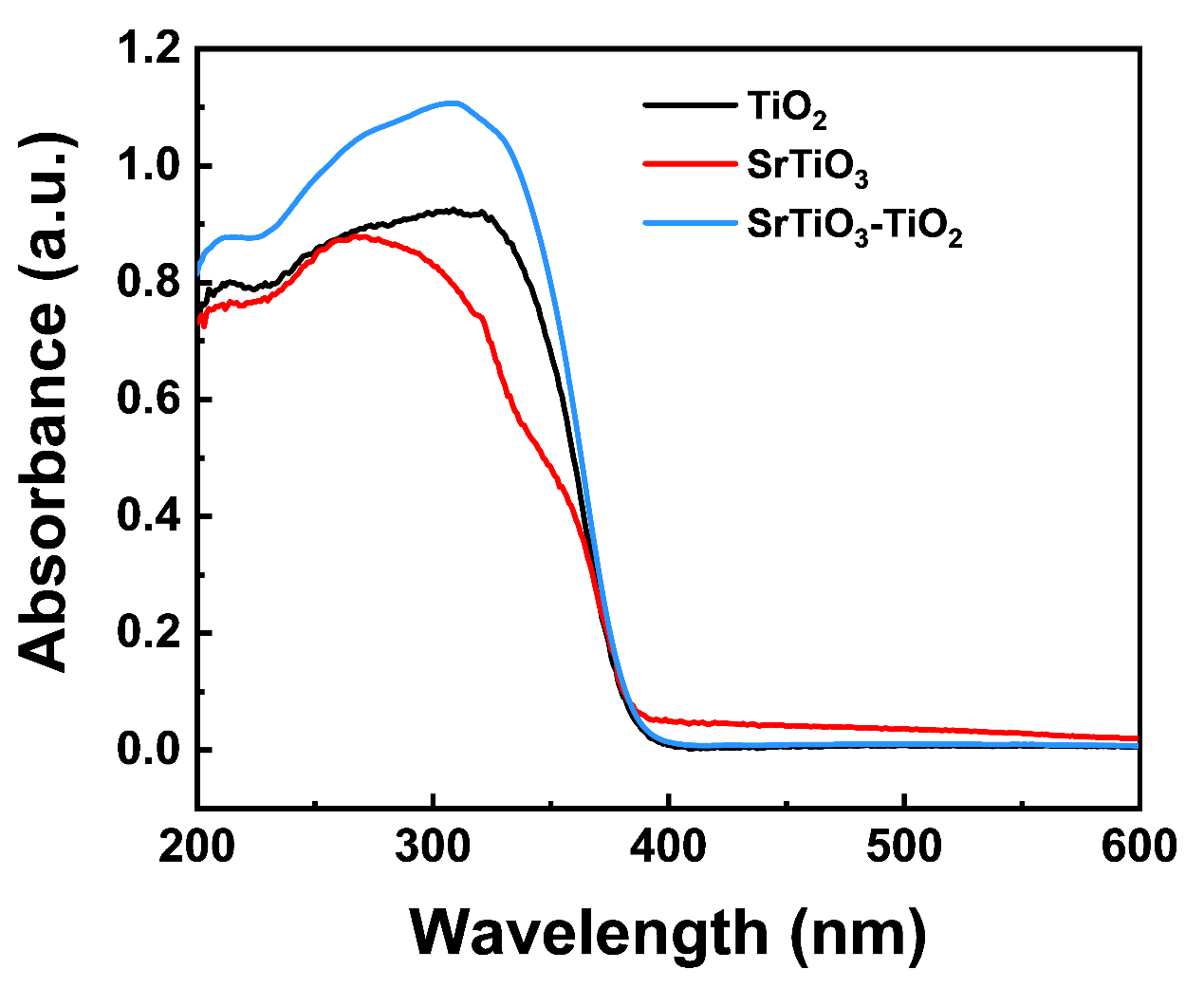
_**Figure S14.** UV-vis absorption spectra of TiO_2_ NRs, SrTiO_3_ NPs and SrTiO_3_-TiO_2_ heterojunction.


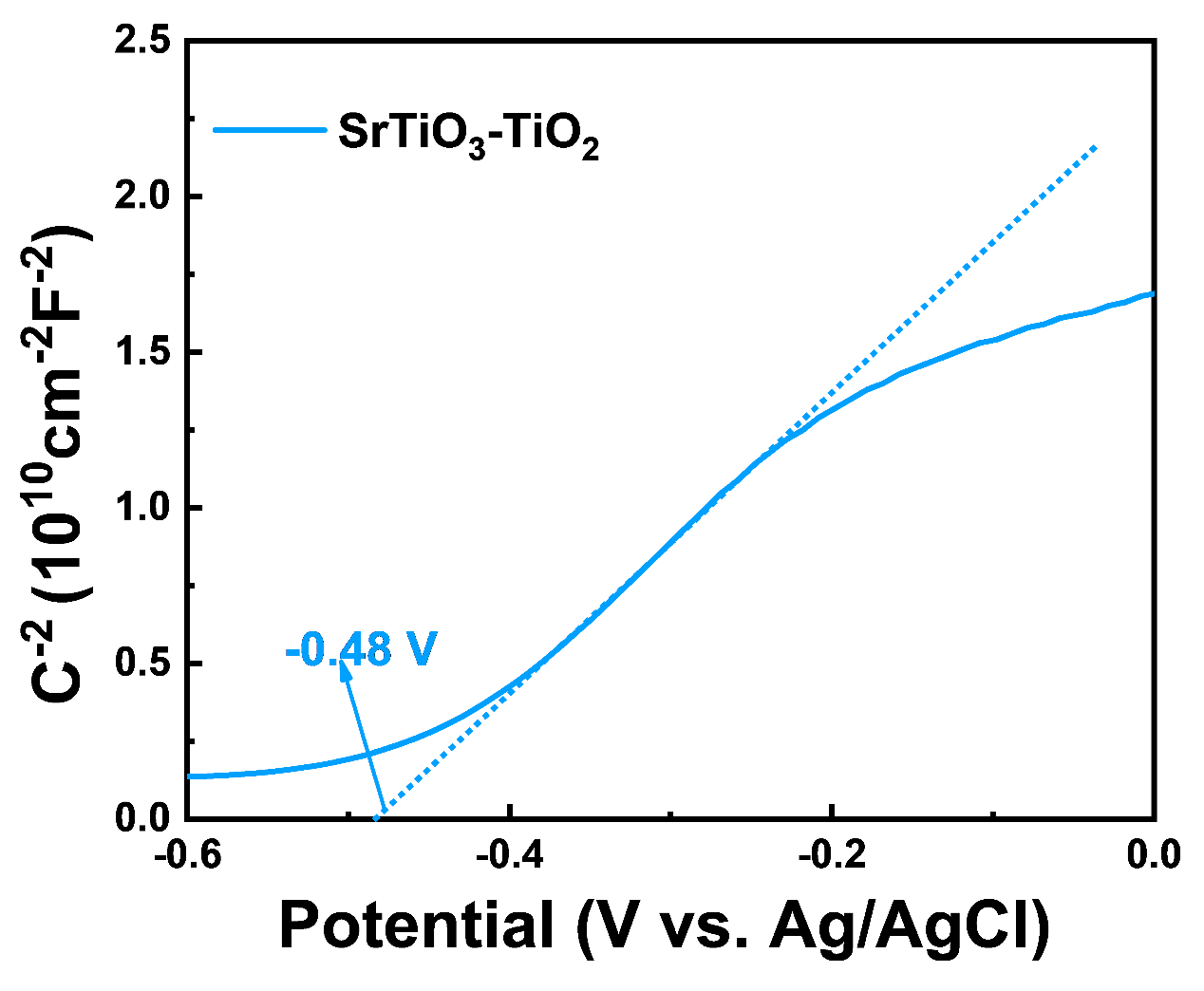


**Figure S15.** Mott-Schottky curves of SrTiO_3_-TiO_2_ heterojunction.

# References

[1] M. Rooman, R. Wintjens, Sequence and conformation effects on ionization potential and charge distribution of homo-nucleobase stacks using M06-2X hybrid density functional theory calculations, J. Biomol. Struct. Dyn., 32 (2013) 532–545.

[2] B. Wang, Y. Ding, X. Tian, Benchmarking model chemistry composite calculations for vertical ionization potential of molecular systems, Chin. Chem. Lett., 36 (2025) 109721.
