# Supplementary material for: Acceleration of Singlet Oxygen Evolution by Sonopiezoelectric Charge Transfer Over SrTiO3‐TiO2 Heterojunction for Selective Oxidation
Source: Exploration (Beijing). 2026 May 28;6(3):20250012. doi: 10.1002/EXP.20250012 (PMC13317800; doi:10.1002/EXP.20250012)
Supplement: Supplementary file 2 — Supporting File 2: exp270174‐sup‐0002‐SuppMat.pptx. [file EXP2-6-20250012-s002.pptx]

## Slide 1
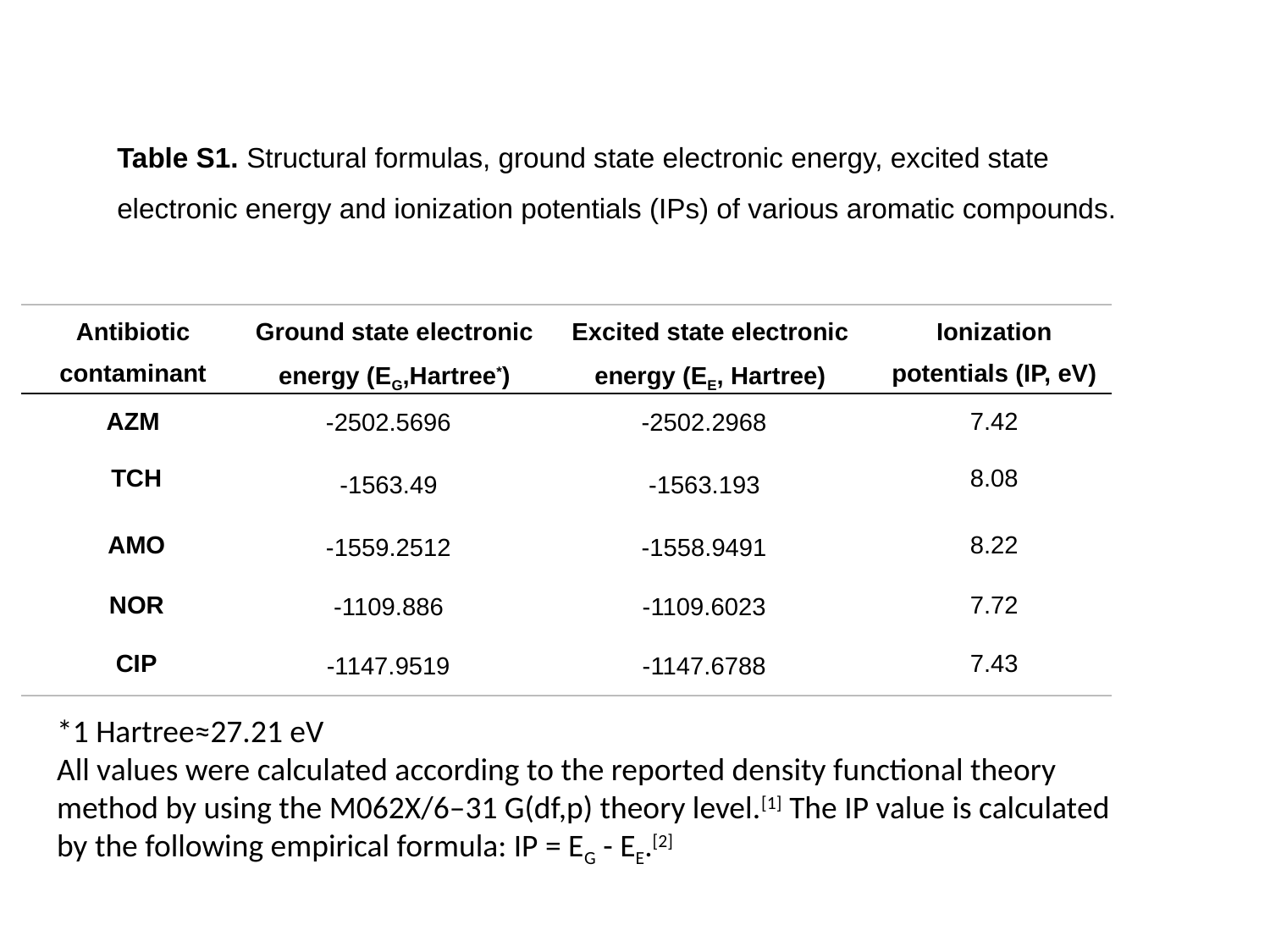

Table S1. Structural formulas, ground state electronic energy, excited state electronic energy and ionization potentials (IPs) of various aromatic compounds.
| Antibiotic contaminant | Ground state electronic energy (EG,Hartree\*) | Excited state electronic energy (EE, Hartree) | Ionization potentials (IP, eV) |
| --- | --- | --- | --- |
| AZM | -2502.5696 | -2502.2968 | 7.42 |
| TCH | -1563.49 | -1563.193 | 8.08 |
| AMO | -1559.2512 | -1558.9491 | 8.22 |
| NOR | -1109.886 | -1109.6023 | 7.72 |
| CIP | -1147.9519 | -1147.6788 | 7.43 |
*1 Hartree≈27.21 eV
All values were calculated according to the reported density functional theory method by using the M062X/6–31 G(df,p) theory level.[1] The IP value is calculated by the following empirical formula: IP = EG - EE.[2]
